# Integrative molecular and clinical modeling of clinical outcomes to PD1 blockade in patients with metastatic melanoma

**DOI:** 10.1038/s41591-019-0654-5

**Published:** 2019-12-02

**Authors:** David Liu, Bastian Schilling, Derek Liu, Antje Sucker, Elisabeth Livingstone, Livnat Jerby-Arnon, Lisa Zimmer, Ralf Gutzmer, Imke Satzger, Carmen Loquai, Stephan Grabbe, Natalie Vokes, Claire A. Margolis, Jake Conway, Meng Xiao He, Haitham Elmarakeby, Felix Dietlein, Diana Miao, Adam Tracy, Helen Gogas, Simone M. Goldinger, Jochen Utikal, Christian U. Blank, Ricarda Rauschenberg, Dagmar von Bubnoff, Angela Krackhardt, Benjamin Weide, Sebastian Haferkamp, Felix Kiecker, Ben Izar, Levi Garraway, Aviv Regev, Keith Flaherty, Annette Paschen, Eliezer M. Van Allen, Dirk Schadendorf

**Affiliations:** 1 Dana-Farber Cancer Institute, Boston, MA USA; 2 Broad Institute of Harvard and MIT, Cambridge, MA USA; 3 Department of Dermatology, University Hospital Würzburg, Würzburg, Germany; 4 Department of Dermatology, University Hospital, Essen, Germany; 5 German Cancer Consortium of Translational Cancer Research, German Cancer Research Center, Heidelberg, Germany; 6 Harvard Medical School, Boston, MA USA; 7 Skin Cancer Center Hannover, Department of Dermatology and Allergy, Hannover Medical School, Hannover, Germany; 8 Department of Dermatology, University Medical Center, Mainz, Germany; 9 Biophysics Program, Harvard University, Cambridge, MA USA; 10 First Department of Medicine, National and Kapodistrian University of Athens, Athens, Greece; 11 Department of Dermatology, University Hospital Zürich, Zürich, Switzerland; 12 Department of Dermatology, University Medical Center Mannheim, Ruprecht-Karl University of Heidelberg, Mannheim, Germany; 13 Skin Cancer Unit, German Cancer Research Center, Heidelberg, Germany; 14 Department of Medical Oncology, The Netherlands Cancer Institute, Amsterdam, the Netherlands; 15 Skin Cancer Center at the University Cancer Centre, Department of Dermatology, Faculty of Medicine, University Hospital Carl Gustav Carus, Technische Universität Dresden, Dresden, Germany; 16 National Center for Tumor Diseases, Dresden, Germany; 17 German Cancer Research Centre, Heidelberg, Germany; 18 Department of Dermatology, Medical Center- University of Freiburg, Faculty of Medicine, University of Freiburg, Freiburg, Germany; 19 Medizinische Klinik III, Klinikum Rechts der Isar, Technische Universität München, Munich, Germany; 20 Department of Dermatology, University Medical Center Tübingen, Tübingen, Germany; 21 Department of Dermatology, University Hospital Regensburg, Regensburg, Germany; 22 Department of Dermatology, University Hospital Berlin, Berlin, Germany; 23 Eli Lilly and Co., Indianapolis, IN USA; 24 Massachusetts General Hospital, Boston, MA USA

**Keywords:** Cancer genomics, Melanoma, Computational biology and bioinformatics

## Abstract

Immune-checkpoint blockade (ICB) has demonstrated efficacy in many tumor types, but predictors of responsiveness to anti-PD1 ICB are incompletely characterized. In this study, we analyzed a clinically annotated cohort of patients with melanoma (*n* = 144) treated with anti-PD1 ICB, with whole-exome and whole-transcriptome sequencing of pre-treatment tumors. We found that tumor mutational burden as a predictor of response was confounded by melanoma subtype, whereas multiple novel genomic and transcriptomic features predicted selective response, including features associated with MHC-I and MHC-II antigen presentation. Furthermore, previous anti-CTLA4 ICB exposure was associated with different predictors of response compared to tumors that were naive to ICB, suggesting selective immune effects of previous exposure to anti-CTLA4 ICB. Finally, we developed parsimonious models integrating clinical, genomic and transcriptomic features to predict intrinsic resistance to anti-PD1 ICB in individual tumors, with validation in smaller independent cohorts limited by the availability of comprehensive data. Broadly, we present a framework to discover predictive features and build models of ICB therapeutic response.

## Abstract (web summary)

Analysis of fully clinically annotated and sequenced melanoma tumor samples collected before anti-PD1 treatment suggests that determinants of response differ on the basis of previous anti-CTLA4 therapy, and that tumor mutational burden may not be a strong predictor of response across melanoma subtypes.

## Main

While ICB has resulted in durable clinical response in multiple tumor types^1–7^, only a subset of patients respond, and predictors of response are not fully characterized. Both tumor-intrinsic and tumor-extrinsic biomarkers of response and resistance to ICB in melanoma have been proposed, including tumor mutational burden (TMB) and neoantigen load^8–11^, immunohistological detection of PD-L1 and CD8^12^ and genetic alterations affecting antigen presentation^13,14^, interferon (IFN)-γ signaling pathways^15^, alternative survival and proliferation pathways^13,16,17^ and aneuploidy^18,19^. Gene expression signatures expressed in tumors^20^ and the tumor immune microenvironment^21^ have also been implicated. However, these observations have often been made in preclinical models or in small clinical cohorts without validation in larger, independent cohorts of patients with melanoma. Furthermore, whether these observations are exclusive to a specific ICB regimen (that is, anti-PD1, anti-CTLA4 or a combination of these) is incompletely characterized. Broadly, the expanding suite of pathways that has been invoked to mediate selective ICB response in melanoma indicates that integrated systems biology models to predict response and survival are necessary, but these have yet to be well developed.

Clinically, the optimal role of anti-CTLA4 in conjunction^3^ or sequentially^22^ with anti-PD1 ICB is unclear. Understanding the differential biology underlying the response to anti-PD1 ICB in tumors with and without previous anti-CTLA4 therapy may inform the rational design of combination therapies and optimize therapy selection for individual patients.

Thus, we performed an integrative study employing genomic, transcriptomic and clinical data from a comprehensively clinically annotated and sequenced cohort of 144 patients with advanced melanoma undergoing anti-PD1 ICB with and without previous anti-CTLA4 ICB to discover biomarkers of response and resistance, and develop clinically applicable parsimonious predictive models.

## Results

### Genomic and clinical cohort characteristics and melanoma subtypes

**Fig. 1:**
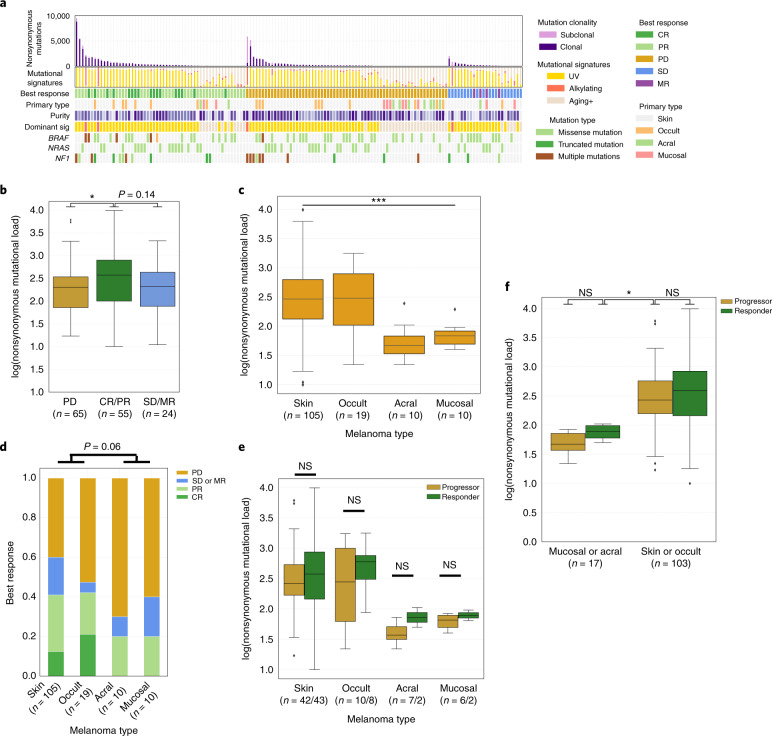
Cohort genomic and clinical characteristics and association of TMB with response. **a**, CoMut plot showing association between clinical and genomic characteristics. Each column represents a tumor. Tumors are ordered by best RECIST criteria response (CR, PR, PD, SD or MR), and within each response subgroup by decreasing nonsynonymous (Nonsyn) mutational load (top row). Nonsynonymous mutational burden is further subdivided into clonal (purple) and subclonal (light purple) mutational load. Mutational signatures (sig) refer to the inferred relative contribution of UV-induced mutations, alkylating DNA damage process and other mutational signatures (aging+). The primary type of melanoma (skin, occult, acral or mucosal) is indicated. Tumor purity is the inferred proportion of the tumor sample that is from cancer cells compared to other cell types (Methods). The dominant mutational signature (that is, the mutational signature associated with the highest proportion of mutations) is indicated. Mutations in *BRAF*, *NRAS* and *NF1* are shown for each tumor. **b**, Mutational load (mut load) in progressors (*n* = 65 patients), responders (*n* = 55 patients) and patients with SD and MR (*n* = 24 patients). Nonsynonymous mutational load is higher in responders (CR and PR) than in progressors (two-sided MWW, *P* = 0.026), but is not significantly different between responders and patients having SD or MR as the best RECIST response (two-sided MWW, *P* = 0.14). **c**, Mutational load by melanoma type. Different melanoma types have different mutational loads (Kruskal–Wallis, *P* = 2.4 × 10^−^^5^): mutational load is higher in cutaneous and occult melanomas (*n* = 124 patients) than in acral and mucosal melanomas (*n* = 20 patients; median 297.5 versus 58; two-sided MWW, *P* = 1.1 × 10^−6^). **d**, Response to anti-PD1 ICB by melanoma type. Cutaneous and occult melanomas (*n* = 124 patients) have higher response rates (~40% CR and PR) versus acral and mucosal melanomas (*n* = 20 patients, 20%; two-sided Fisher’s exact test, *P* = 0.06). **e**, Mutational load in responders versus progressors stratified by melanoma type. There was no significant difference between responder and progressor mutational loads when melanomas were stratified by type (two-sided MWW; progressors versus responders (PD/R): skin (*n* = 42/43), *P* = 0.27; occult (*n* = 10/8), *P* = 0.35; acral (*n* = 7/2), *P* = 0.19; mucosal (*n* = 6/2), *P* = 0.40). Mutational load was also not a significant predictor of response in combined logistic regression after adjusting for melanoma type (*P* = 0.24). **f**, TMB in responders versus nonresponders, stratified by skin or occult melanomas versus mucosal or acral melanomas. Within each subgroup, responders trended toward having higher TMB than nonresponders (cutaneous/occult (*n* = 52 progressors and 51 responders): MWW, *P* = 0.14; mucosal/acral (*n* = 13 progressors and 4 responders): MWW, *P* = 0.08). Notably, responders with mucosal or acral melanoma (*n* = 4) had a lower mutational load than progressors with cutaneous or occult melanoma (*n* = 52; MWW, *P* = 0.03). Boxplots: box limits indicate the IQR (25th to 75th percentiles), with a center line indicating the median. Whiskers show the value ranges up to 1.5 × IQR above the 75th or below the 25th percentiles, with outliers beyond those ranges shown as individual points. *P < 0.05, **P < 0.01, ***P < 0.001. NS, not significant.

**Extended Data Fig. 1:**
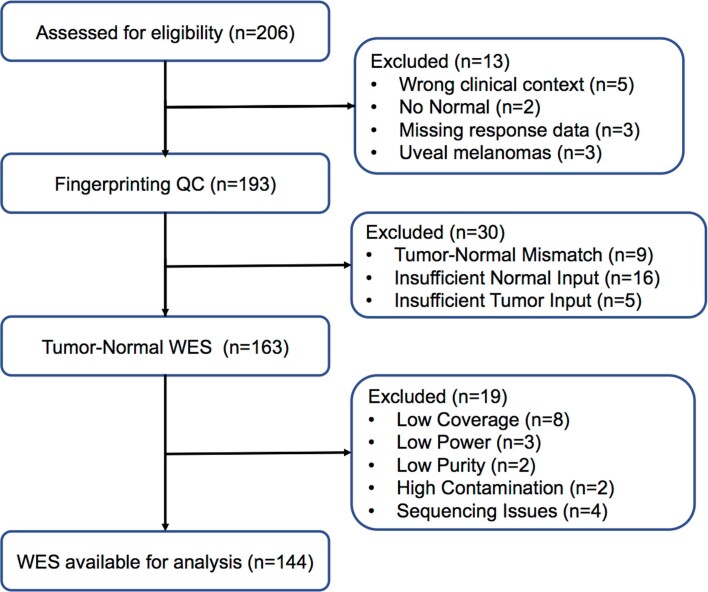
Consort Diagram showing inclusion, exclusion, and quality control criteria for patients/tumors included in analysis.

We identified 206 patients diagnosed with advanced melanoma and treated with anti-PD1 ICB, and performed whole-exome sequencing (WES) on matched pretreatment tumor samples and normal tissue^23^, and whole-transcriptome sequencing (RNA-seq) on available pretreatment tumor tissue. After quality control (Methods), WES data from 144 patients and RNA-seq data from 121 patients were available for final evaluation (Extended Data Fig. 1). Best objective response (BOR) to anti-PD1 ICB using RECIST (v.1.1) criteria (Methods) included 45% with progressive disease (PD), 14% with stable disease (SD), 3% with mixed response (MR), 26% with partial response (PR) and 12% with complete response (CR; Fig. 1a), for an overall response rate of 38%. Overall, 73% were cutaneous melanomas, 13% were of occult origin, 7% were mucosal and 7% were acral in origin. A total of 44% (*n* = 64) of patients had previous treatment with ipilimumab, whereas 56% (*n* = 80) were naive to ipilimumab. The median follow-up for survival was 29.9 months. Other clinical characteristics are detailed in Table 1.

**Table 1:** Cohort clinical characteristics

	*n* (%)
Total cohort	144 (100)
Drug received	
Nivolumab	59 (41.0)
Pembrolizumab	85 (59.0)
Sex	
Female	60 (41.7)
Male	84 (58.3)
Stage	
Unresectable stage III	10 (6.9)
M1a	8 (5.6)
M1b	18 (12.5)
M1c	108 (75.0)
Active brain metastases	
Yes	16 (11.1)
No	128 (88.9)
Elevated LDH	
Yes	71 (49.3)
No	70 (48.6)
Unknown	3 (2.1)
ECOG performance status	
0	99 (68.8)
1	37 (25.7)
2	2 (1.4)
3	1 (0.7)
Unknown	5 (3.5)
Primary melanoma	
Cutaneous	105 (72.9)
Occult	19 (13.2)
Acral	10 (6.9)
Mucosal	10 (6.9)
Received anti-PD1 ICB	
First line	71 (49.3)
Second line or later	73 (50.7)
Previous ipilimumab	
Yes	60 (41.7)
No	84 (58.3)

The number of patients with the given characteristic is shown, with the number in parentheses indicating the percentage of patients represented. ECOG, Eastern Cooperative Oncology Group.

Overall the median nonsynonymous TMB was 6.5 mutations per Mb (250.5 mutations per exome), with an interquartile range (IQR) of 2.0–14.4 mutations per Mb (77.75–578.5 mutations per exome). Overall, 39% of tumors had *BRAF* mutations, 30% had *NRAS* mutations and 17% had *NF1* mutations (Fig. 1a). The median tumor purity (the proportion of sample DNA from tumor cells) was 0.67 (IQR 0.46–0.83) and the median tumor heterogeneity (the proportion of subclonal mutations) was 0.17 (IQR 0.12–0.25). The median purity-corrected tumor ploidy (Methods) was 2.15 (IQR 2.01–3.12), with 38% of tumors inferred to have genome doubling, consistent with previous reports^24^. The predominant mutational signature in most tumors was related to ultraviolet (UV) exposure^25^ (69% related to UV, 3% related to alkylating chemotherapy^25^ and 28% related to another predominant mutational signature, mostly associated with aging^25^; Fig. 1a). Individual tumor characteristics are detailed in Supplementary Table 1.

**Extended Data Fig. 2:**
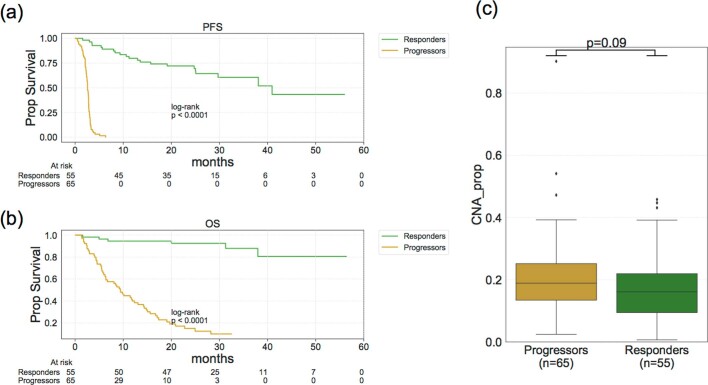
Survival and genomic copy number characteristics of responders (n = 55 patients) (defined as CR or PR as best response) vs. progressors (n = 65 patients) (defined as PD as best response). (a) PFS Kaplan Meier survival curves; two-sided log-rank p = 2.1e-28 (b) OS Kaplan Meier survival curves; two-sided log-rank p = 5.1e-18 (c) Proportion of tumor genome with copy number alterations (two-sided MWW p = 0.09). Boxplots: Box limits indicate the interquartile range (25th-75th percentile), with a center line indicating the median. Whiskers show the value ranges up to 1.5xIQR above the 75th or below the 25th percentile, with outliers beyond those ranges shown as individual points. PD = progressive disease. CR = complete response. PR = partial response.

To discover differential features associated with response, we compared clinical responders (*n* = 55) to progressors (*n* = 65), excluding patients with SD (*n* = 20) and MR (*n* = 4) as the BOR. Overall survival (OS) and progression-free survival (PFS) were significantly different between these groups (log-rank *P* < 0.00001 for both comparisons; Extended Data Fig. 2a,b).

TMB was higher in responders than in progressors (Mann–Whitney–Wilcoxon (MWW), *P* = 0.026; Fig. 1b), but there was substantial overlap between responders and progressors. We hypothesized that the relationship between response and TMB might further be confounded by melanoma subtype. TMB was significantly different between different melanoma subtypes (Kruskal–Wallis, *P* = 2.4 × 10^−5^; Fig. 1c), with cutaneous and occult melanomas having similar and higher TMB than acral and mucosal melanomas^26^ (median of 297.5 versus 58, nonsynonymous mutations; MWW, *P* = 1.1 × 10^−6^), with a higher response rate (~40% versus ~20%; Fisher’s exact test, *P* = 0.06; Fig. 1d). When stratified by melanoma subtype, responders did not have significantly higher TMB than nonresponders (Fig. 1e), and, in multivariate logistic regression adjusting for melanoma subtype, TMB was not a significant predictor (*P* = 0.24). Strikingly, responders with mucosal or acral melanoma had a lower TMB than progressors with cutaneous or occult melanoma (MWW, *P* = 0.03; Fig. 1f), suggesting that disease subtype confounds the association between TMB and response to anti-PD1 therapy.

### Genomic and transcriptomic features associated with response

**Fig. 2:**
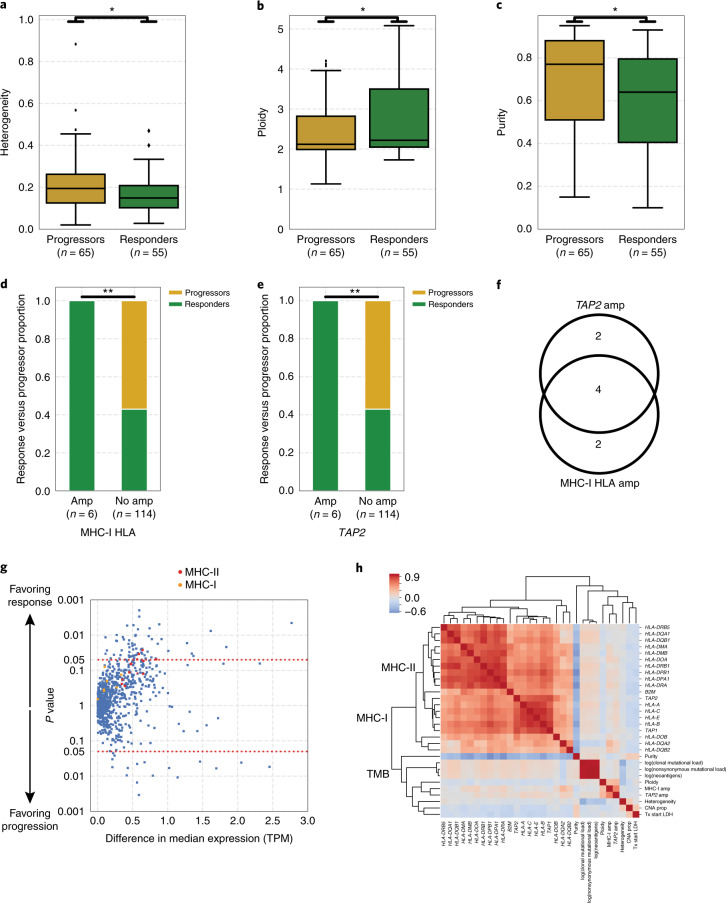
Genomic and transcriptomic features associated with response. All *P* values are unadjusted, unless otherwise indicated. **a**, Tumor heterogeneity, defined as the proportion of subclonal mutations in each tumor (Methods), in responders (CR or PR) versus progressors (PD). Progressors (*n* = 65 patients) had greater heterogeneity than responders (*n* = 55 patients; two-sided MWW, *P* = 0.02). **b**, Tumor ploidy, defined as the overall genomic copy number (a normal diploid cell has a copy number of 2; Methods), in responders versus progressors. Responders (*n* = 55 patients) had higher tumor ploidy than progressors (*n* = 65 patients; two-sided MWW, *P* = 0.04). **c**, Tumor purity, defined as the proportion of DNA from tumor versus other cells in the sample (Methods), in responders versus progressors. Progressors (*n* = 65 patients) had higher tumor purity than responders (*n* = 55 patients; two-sided MWW, *P* = 0.04). **d**, Response versus progression in *TAP2-*amplified tumors versus other tumors. *TAP2* amplification (*n* = 6 patients) was associated with response (two-sided Fisher’s exact test, *P* = 0.008). **e**, Response versus progression in tumors with amplified MHC-I HLA genes (*HLA-A*, *HLA-B* or *HLA-C*) versus other tumors. MHC-I HLA amplification (*n* = 6 patients) was associated with response (two-sided Fisher’s exact test, *P* = 0.008). **f**, Venn diagram showing the overlap of *TAP2*-amplified tumors and tumors with amplification of MHC-I HLA genes. Four tumors had amplifications on chromosome 6, including the MHC-I genes *HLA-A*, *HLA-B*, *HLA-C* and *TAP2* and two tumors each had amplifications in one but not the other region, for a total of eight tumors with amplifications in either. **g**, Difference in the median expression and two-sided MWW *P* value of association between 938 immune-related genes^56^ and features in responders versus progressors. Expression levels of MHC-II HLA genes (red), MHC-I HLA genes and antigen-presentation machinery (APM)-related genes (orange) are shown. **h**, Hierarchical clustering of the correlation matrix between genomic, clinical and transcriptomic features associated with response. Color indicates the Pearson correlation between features, from perfect negative correlation (Pearson, *r* = −1, blue) to perfect positive correlation (Pearson, *r* = 1, red). An immune-related cluster of MHC-I- and MHC-II-related gene expression is observed, with subclusters of MHC-I and MHC-II genes. Mutational and neoantigen load are highly correlated and form a cluster independently from the immune cluster (for example, Pearson correlation, *r* = 0.15, *P* = 0.11 between ssGSEA of MHC-II HLA genes and nonsynonymous mutational load). Purity is negatively correlated with the immune cluster and is independent of ploidy and heterogeneity. The sample size for each correlation depended on the number of available data points: correlations involving exclusively genomic or clinical data had *n* = 144 tumor samples, whereas correlations involving transcriptomic features had *n* = 121 tumor samples with data available. Tx, treatment. Boxplots: box limits indicate the IQR (25th to 75th percentile), with a center line indicating the median. Whiskers show the value ranges up to 1.5 × IQR above the 75th or below the 25th percentile, with outliers beyond those ranges shown as individual points. *P < 0.05, **P < 0.01, ***P < 0.001; NS, not significant.

Higher tumor purity and heterogeneity were associated with progression (MWW, *P* = 0.04 and *P* = 0.02, respectively; Fig. 2a,c), whereas ploidy was lower in progressors (MWW, *P* = 0.04; Fig. 2b). The proportion of the tumor genome with copy number alterations (CNAs) trended toward being higher in patients with PD (MWW, *P* = 0.09; Extended Data Fig. 2c).

**Extended Data Fig. 3:**
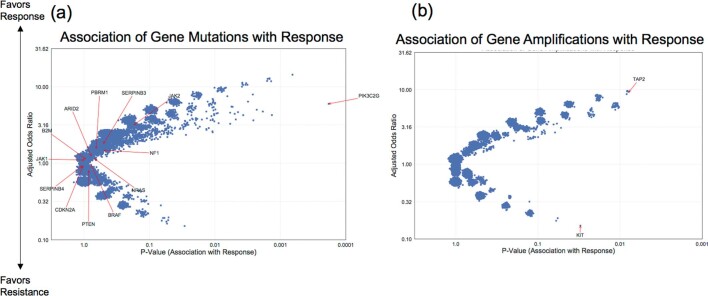
Association of Genomic Alterations in Genes with Response vs. Progression. For each gene, the association of a genomic alteration with response was tested using a two-sided Fisher’s Exact test, and the odds ratio (OR) and p-value calculated. The adjusted OR is a conservative estimate generated by adding 1 to both mutant gene responders and progressors, thus moving the OR estimate closer to 1. The x- and y-axis are on log scales, but labeled in the original units. (**a**) Association of gene mutations with response (n = 55 patients) vs. progression (n = 65 patients). (**b**) Association of gene amplifications with response (n = 55 patients) vs. progression (n = 65 patients).

Given these observations, we performed an unbiased analysis for single-gene predictors of response to anti-PD1 ICB across all mutated genes detected in this cohort. After multiple-hypothesis test correction, no genes were significantly associated with response or resistance to therapy (Extended Data Fig. 3a and Supplementary Table 2), highlighting the large sample sizes needed for adequate power to detect these associations^27^. We observed only rare mutations in major histocompatibility complex class I (MHC-I) antigen-presentation genes (*TAP1*, *TAP2*, *B2M*, *HLA-A*, *HLA-B* and *HLA-C*)^14,28^. Mutations in *SERPINB3* or *SERPINB4*^29^ were not associated with response *(*Fisher’s exact test, *P* = 0.51 and *P* = 1.0, respectively). Loss of heterozygosity (LOH) in *B2M*^14^ was found in 9 of 55 (16%) responders and 16 of 65 (25%) progressors (odds ratio (OR) = 0.6) but was not significantly associated with resistance (Fisher’s exact test, *P* = 0.37). LOH in *HLA-A*, *HLA-B* or *HLA-C*^28^ was not associated with response or resistance to therapy (Fisher’s exact test, *P* = 0.52, *P* = 0.57 and *P* = 0.84, respectively). Confirming previous findings, LOH of *JAK1*^13,30,31^ was associated with resistance (OR = 0.33; Fisher’s exact test, *P* = 0.02). Biallelic *CDKN2A* alteration^27^ was found in 15 of 55 (27%) responders and 25 of 65 (38%) progressors (OR = 0.6), but was not significant (Fisher’s exact test, *P* = 0.24).

**Extended Data Fig. 4:**
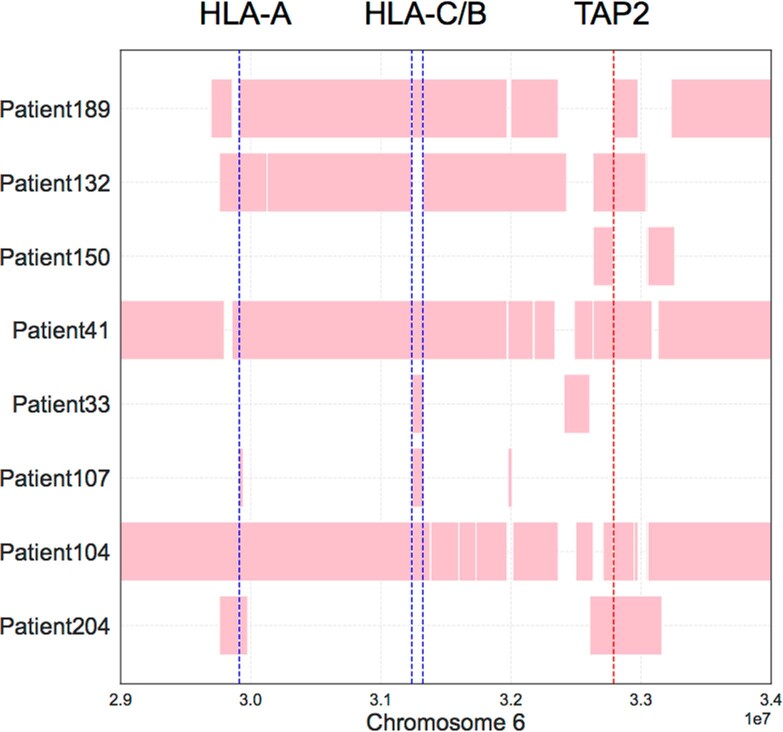
Amplification of Chromosome 6 Regions in TAP2, HLA-A/B/C amplified tumors. Patients with amplifications in TAP2 or HLA-A/B/C are shown, with inferred amplifications in the relevant section of Chromosome 6 shown. 6 patients had amplifications in TAP2, 6 patients had amplifications in HLA-A/B/C, and 4 patients had amplifications of a chromosomal region including all four genes.

We also performed an unbiased analysis of the association between focal gene amplifications and response to therapy. While no gene amplification was significant after multiple-hypothesis test correction (Extended Data Fig. 3b and Supplementary Table 3), amplification of *TAP2*, an integral part of the MHC-I antigen-loading pathway, was found exclusively in responders to therapy (*n* = 6; Fisher’s exact test, *P* = 0.008; Fig. 2d). *TAP2* is located at 6p21 in a region encoding both MHC-I and MHC-II human leukocyte antigen (HLA) loci, and four out of six *TAP2* amplifications were associated with larger amplifications across the region, while two out of six amplifications were more focal (Extended Data Fig. 4). Notably, tumors with amplifications in this region encompassing the MHC-I-related *HLA-A*, *HLA-B* and *HLA-C* genes (a region of approximately 1.5 Mb; *n* = 6) were also associated with response to therapy (Fisher’s exact test, *P* = 0.008; Fig. 2e), with four out of six also having amplifications in *TAP2*. Altogether, eight patients had amplification of either *TAP2* or *HLA-A*, *HLA-B* or *HLA-C* amplification (Fig. 2f and Extended Data Fig. 4), and were exclusively responders (Fisher’s exact test, *P* = 0.001).

We then examined the expression of antigen-presentation molecules and their association with response. Interestingly, expression of all 13 MHC-II-associated HLA genes was higher in responders (collective two-sided binomial test, *P* = 0.0002; Fig. 2g), with four genes (*HLA-DMA*, *HLA-DMB*, *HLA-DOB* and *HLA-DOB*) individually passing a statistical significance threshold (MWW, *P* < 0.05; Supplementary Table 4). MHC-I antigen-presentation genes all trended toward having higher expression in responders (collective two-sided binomial test, *P* = 0.02; Fig. 2g and Supplementary Table 4), but none passed the statistical significance threshold.

To examine pathways differentially enriched in responders versus progressors, we next performed unbiased gene set enrichment analysis (GSEA^32^; Methods) using the Hallmark gene sets^33^. A total of 24 pathways were enriched (false discovery rate (FDR), *q* < 0.1) in responders, and 5 of the top 6 enriched pathways were immune related, including IFN-γ response, genes involved in allograft rejection, complement, the inflammatory response and interleukin (IL)-6–JAK–STAT3 signaling (Supplementary Table 5). No pathways were significantly enriched in progressors.

We further evaluated various transcriptomic signatures^18,19,21,34–40^ that had been proposed and demonstrated in various settings to be associated with response to immunotherapy (Methods), but we found no significant differences (*P* < 0.05) in these signatures between responders and progressors within our cohort (Supplementary Table 6).

Immune infiltrate has been associated with response to immunotherapy across multiple cancer types and immune therapies^12,34^. We inferred the absolute level of immune infiltrate within each sample using an immune deconvolution algorithm (CIBERSORT^41^ using the LM22 signature matrix). We found no significant difference in the total immune infiltrate or abundance of individual immune cell subsets in responders versus progressors (Supplementary Table 7). We also generated profiles of expression of immune cell subset signatures derived from single-cell analyses^42^ and found that the expression of multiple signatures was significantly higher (unadjusted MWW, *P* < 0.05) in responders versus progressors, including for signatures of overall immune infiltrate, T cells, B cells, macrophages, CD8^+^ cytotoxic exhausted T cells and CD4^+^ exhausted T cells (Supplementary Table 7). Although the strength of association differed by deconvolution method, both approaches generally agreed on the direction of association, providing evidence for a moderate association of immune infiltrate with the response to anti-PD1 ICB.

### Correlations between molecular features

**Extended Data Fig. 5:**
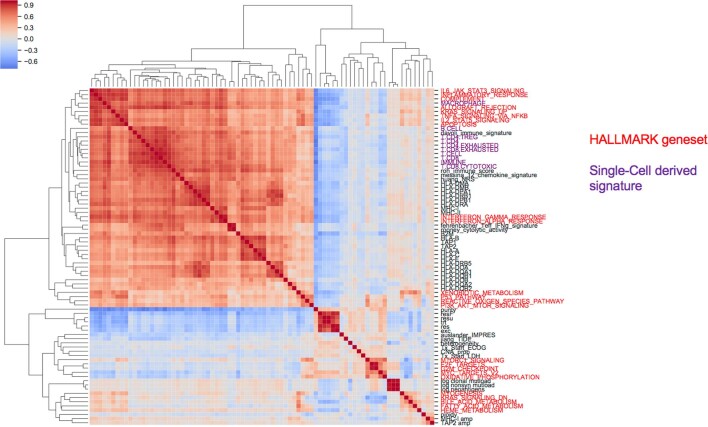
Hierarchical clustering of the correlation matrix between transcriptional signatures previously associated with immunotherapy response and hallmark gene-sets, genomic, clinical, and transcriptomic features associated with response in our cohort. The color indicates the Pearson correlation between features, from perfect negative correlation (Pearson r = -1, blue) to perfect positive correlation (Pearson r = 1, red). Most previously hypothesized signatures cluster within an immune-activity related group with immune-related gene-sets. Sample size for each correlation depends on the number of available data points: correlations involving exclusively genomic or clinical data had n = 144 tumor samples, while correlations involving transcriptomic features had n = 121 tumor samples with data available.

To understand the relationship between predictors of response, we performed hierarchical clustering of the correlation coefficients between associated predictors (Fig. 2h). Clustered features are correlated and may reflect the same underlying biology, whereas separate clusters may reflect independent feature categories. A large immune-related cluster with MHC-II- and MHC-I-related subclusters was observed, with a separate independent cluster of mutation- and neoantigen-load-related features, suggesting independent feature categories. Tumor purity was negatively correlated with the immune cluster, suggesting that low tumor purity may be a proxy for higher immune infiltrates within the tumor sample. Other features, including tumor heterogeneity and ploidy, were independent from these two clusters. Extending this analysis to previously hypothesized signatures and Hallmark gene set signatures (Extended Data Fig. 5), immune activity signatures, including signatures of cytolytic^21^ and cytotoxic^19^ activity, IFN-γ and T effector cells^18,35^, immune chemokines^38^ and single-cell-derived immune cell signatures^42^, clustered together. A tumor-intrinsic resistance program signature^42^, signatures of T cell dysfunction and exclusion^37^ and comparative immune-checkpoint gene expression^34^ were distinct from the immune cluster. Overall, these findings suggested that multiple previously hypothesized predictors of ICB response reflect the same underlying biological state and additional independent classes of predictors exist that may provide additional predictive power.

### Previous exposure to anti-CTLA4 ICB

**Fig. 3:**
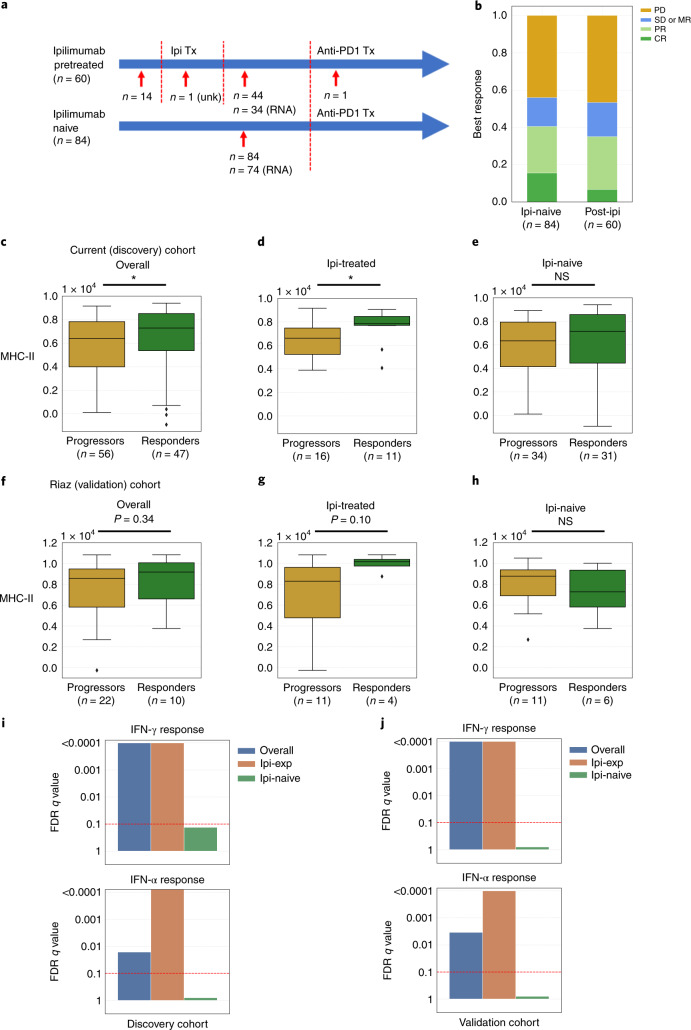
Differential predictors of response and progression in ipilimumab-treated tumors versus ipilimumab-naive tumors. **a**, Timeline showing when sequenced biopsies were obtained from tumors that were treated with ipilimumab or naive to ipilimumab in the course of therapy. Subsequent analyses focused on comparing tumor biopsies obtained after ipilimumab treatment (*n* = 45 WES, *n* = 34 RNA-seq) to ipilimumab-naive tumor biopsies (*n* = 84 WES, *n* = 74 RNA-seq). **b**, Best RECIST response by ipilimumab pretreatment status. There was no difference between the distribution of responses in naive (*n* = 84) and pretreated (*n* = 60) patients (two-sided chi-squared test, *P* = 0.44; degrees of freedom (d.f.) = 3). **c**, ssGSEA of MHC-II HLA genes (Methods) in responders (*n* = 47 patients) versus progressors (*n* = 56 patients) in the overall cohort. MHC-II scores were higher in responders than in progressors (two-sided MWW, *P* = 0.03). **d**, ssGSEA of MHC-II HLA genes in responders versus progressors in the post-ipilimumab-treatment subgroup. MHC-II scores were higher in responders (*n* = 11 patients) than in progressors (*n* = 16 patients; two-sided MWW, *P* = 0.03). **e**, ssGSEA of MHC-II HLA genes in responders (*n* = 31 patients) versus progressors (*n* = 34 patients) in the ipilimumab-naive subgroup. There was no significant difference in MHC-II scores between responders and progressors (two-sided MWW, *P* = 0.31). **f**, MHC-II HLA gene set scores (ssGSEA) in responders (*n* = 10 patients) versus progressors (*n* = 22 patients) in a validation cohort (Methods; two-sided MWW, *P* = 0.34). **g**, MHC-II HLA gene set scores (ssGSEA) in responders (*n* = 4 patients) versus progressors (*n* = 11 patients) in the ipilimumab-treated subgroup of a validation cohort (two-sided MWW, *P* = 0.10). **h**, MHC-II HLA gene set scores (ssGSEA) in responders (*n* = 6 patients) versus progressors (*n* = 11 patients) in the ipilimumab-naive subgroup of a validation cohort (two-sided MWW, *P* = 0.80). **i**, Selected Cancer Hallmark gene sets (GSEA) enriched in responders versus progressors in the overall (*n* = 47 responders and 56 progressors), post-ipilimumab-treatment (*n* = 11 responders and 16 progressors), and ipilimumab-naive (*n* = 31 responders and 34 progressors) subgroups of our discovery cohort. IFN-γ and IFN-α response pathways were enriched in responders in the overall (FDR, *q* < 0.001 and *q* = 0.02, respectively) and ipilimumab-treated (*q* < 0.001, both) subgroups but not in the ipilimumab-naive subgroups (*q* = 0.13 and *q* = 0.997, respectively) in the discovery cohort (empiric, *P* = 0.183 and *P* = 0.18, respectively for the difference in *q* values between the subgroups; Methods). **j**, Selected Cancer Hallmark gene sets (GSEA) enriched in responders versus progressors in the overall, post-ipilimumab-treatment and ipilimumab-naive subgroups in an independent validation cohort. IFN-γ and IFN-α response pathways were enriched in responders in the overall (FDR, *q* < 0.0001 and *q* = 0.0034, respectively) and ipilimumab-treated (*q* < 0.0001, both) subgroups but not in the ipilimumab-naive subgroup (*q* = 0.87 and *q* = 0.03 (enriched in progressors), respectively) in the discovery cohort. Pathways favoring enrichment in progressors (as opposed to responders) are visualized here with an FDR *q* value of 1. All Hallmark pathways and their GSEA enrichment scores are shown in Supplementary Table 5. Boxplots: box limits indicate the IQR (25th to 75th percentile), with a center line indicating the median. Whiskers show the value ranges up to 1.5 × IQR above the 75th or below the 25th percentile, with outliers beyond those ranges shown as individual points. *P < 0.05, **P < 0.01, ***P < 0.001. NS, not significant.

Our cohort contained patients with previous exposure to ipilimumab in anti-CTLA4 ICB (*n* = 60) and patients who were naive to ipilimumab (*n* = 84; Fig. 3a). Despite the groups having similar response rates (Fig. 3b), we hypothesized that these two groups might have differential predictors of response and resistance to anti-PD1 ICB. We performed a focused analysis of patients who were treated with ipilimumab and biopsied after treatment (*n* = 44 with WES and *n* = 34 with RNA-seq) versus patients who were naive to ipilimumab (*n* = 84 with WES and *n* = 71 with RNA-seq). A composite, rank-based score of MHC-II HLA expression (single-sample GSEA (ssGSEA)^43^; Methods) was higher in responders than in progressors in the overall cohort and the subgroup treated with ipilimumab, but was not significantly different in the subgroup that was naive to ipilimumab (Fig. 3c–e; MWW, *P* = 0.03, *P* = 0.03 and *P* = 0.31, respectively). We found very similar results in the largest available independent validation cohort^44^ with information on previous ipilimumab treatment (Fig. 3f–h), although the difference was not significant in this smaller cohort (*n* = 32 patients, 15 of whom were treated with ipilimumab and 17 of whom were naive for ipilimumab).

**Extended Data Fig. 6:**
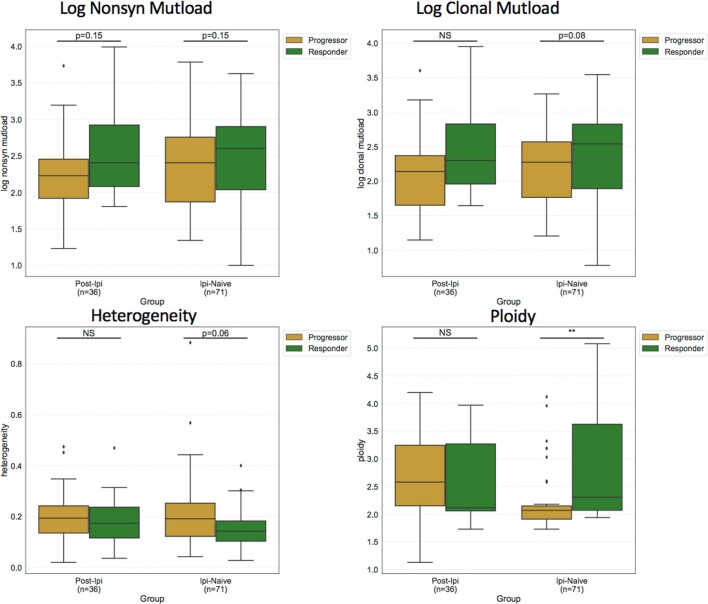
Expression of Genomic Features in Responders vs. Progressors in ipilimumab-treated and -naive subsets. Two-sided Mann-Whitney-Wilcoxon tests were used to compare responders to progressors (ipilimumab-treated: n = 16 responders/20 progressors; ipilimumab-naïve: n = 34 responders/37 progressors). All p-values are unadjusted. Log Nonsyn Mutload: Ipi-treated p = 0.15; Ipi-naïve p = 0.15. Log Clonal Mutload: Ipi-treated p = 0.17; Ipi-naïve p = 0.08. Heterogeneity: Ipi-treated p = 0.51; Ipi-naïve p = 0.057. Ploidy: Ipi-treated p = 0.45; Ipi-naïve p = 0.002. Boxplots: Box limits indicate the interquartile range (25th-75th percentile), with a center line indicating the median. Whiskers show the value ranges up to 1.5xIQR above the 75th or below the 25th percentile, with outliers beyond those ranges shown as individual points.

We next examined the association of TMB, purity, ploidy and heterogeneity with response stratified by previous ipilimumab therapy (Extended Data Fig. 6a). Unlike previous studies^44,45^, we found no specific association of a higher TMB with response in the ipilimumab-naive versus ipilimumab-treated subgroup (MWW, *P* = 0.15, both). However, higher heterogeneity and lower ploidy were associated with progressors only in the ipilimumab-naive subgroup (MWW, *P* = 0.06 and *P* = 0.004, respectively).

We analyzed the differential expression of specific immune-related genes in responders versus progressors in ipilimumab-treated and ipilimumab-naive subgroups and found that higher expression of various immune-related pathways distinguished responders from progressors in ipilimumab-treated but not ipilimumab-naive subgroups (all *P* values are unadjusted). Examples included the leukocyte chemoattractants *CXCL9* and *CXCL10* and their receptor *CXCR3* (MWW, *P* = 0.05, *P* = 0.08 and *P* = 0.02, respectively, in the ipilimumab-treated subgroup), *CD3D* (MWW, *P* = 0.02), B cell markers *CD19* (MWW, *P* = 0.04) and *CD20* (*MS4A1*; MWW, *P* = 0.002) and macrophage marker *CD163* (MWW, *P* = 0.03). Interestingly, *CD4*, *FOXP3* *and CTLA4* also followed this pattern of higher expression in responders in the ipilimumab-treated subgroup *(*MWW, *P* = 0.06, *P* *=* 0.06 and *P* = 0.008, respectively), but *CD8A* and *CD8B* had less evidence of association with response in either ipilimumab-treated (MWW, *P* = 0.17 and *P* *=* 0.27, respectively) or ipilimumab-naive (MWW, *P* = 0.93 and *P* *=* 0.49, respectively) subgroups. Expression of *TAP2* was higher (MWW, *P* = 0.02) in responders than in progressors in the ipilimumab-treated subgroup but not in the ipilimumab-naive subgroup (MWW, *P* = 0.98). In contrast, *TGFB2* expression was higher in progressors in the ipilimumab-naive subgroup (MWW, *P* = 0.002) but not in the ipilimumab-treated subgroup (MWW, *P* = 0.43). The complete set of gene expression comparisons in the overall, ipilimumab-treated and ipilimumab-naive cohorts is in Supplementary Table 4.

We then repeated GSEA to examine the pathways differentially enriched in responders versus progressors between ipilimumab-treated and ipilimumab-naive subgroups. The most differentially enriched pathways were related to immune response: the IFN-γ and IFN-α responses were significantly enriched in responders in the ipilimumab-treated subgroup (FDR, *q* < 0.0001, both), but not in the ipilimumab-naive subgroup (FDR, *q* = 0.13 and *q* = 0.996, respectively; Fig. 3i). Using permutation testing (Methods), we found a nonsignificant empiric *P* value of 0.183 and 0.18, respectively, for this difference in enriched pathways in these subgroups in our discovery cohort. However, we repeated the analysis in an independent validation cohort^46^ and found similar results (Fig. 3j). Complete GSEA results are provided in Supplementary Table 5.

**Fig. 4:**
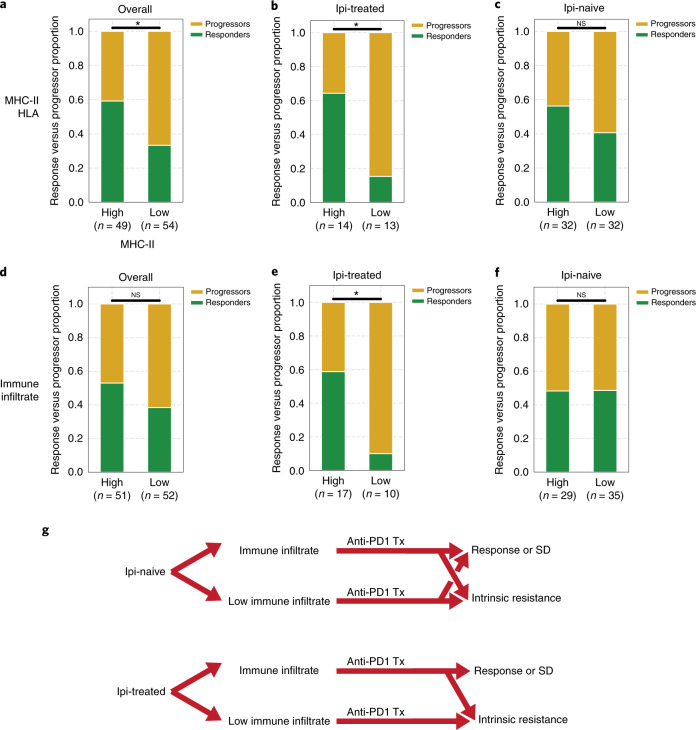
Progression versus response by immune infiltrate and MHC-II HLA expression stratified by ipilimumab treatment. **a**, Proportion of responders versus progressors in subgroups with high and low MHC-II HLA score (ssGSEA; divided by the median). Overall, a high MHC-II HLA score was associated with higher response (MHC-II high: *n* = 29 responders and 20 progressors; MHC-II low: *n* = 18 responders and 36 progressors; two-sided Fisher’s exact test, *P* = 0.01; OR = 2.9, 95% CI 1.3–6.5). **b**, As in **a** but in the ipilimumab-treated subgroup. Tumors with low MHC-II HLA score (ssGSEA) were associated with PD (MHC-II high: *n* = 9 responders and 5 progressors; MHC-II low: *n* = 2 responders and 11 progressors; two-sided Fisher’s exact test, *P* = 0.02; OR = 9.9, 95% CI 1.5–63.7). **c**, As in **a** but in the ipilimumab-naive subgroup. There was no significant difference between tumors with high and low MHC-II HLA scores (ssGSEA; MHC-II high: *n* = 18 responders and 14 progressors; MHC-II low: *n* = 13 responders and 19 progressors; two-sided Fisher’s exact *test, P* = 0.32; OR = 1.9, 95% CI 0.7–5.1). **d**, Proportion of responders versus progressors in subgroups with high and low immune infiltrate scores (divided by the median). Overall, there was no statistically significant difference in the proportion of responders versus progressors in tumors with high versus low immune infiltrate (infiltrate high: *n* = 27 responders and 24 progressors; infiltrate low: *n* = 20 responders and 32 progressors; two-sided Fisher’s exact test, *P* = 0.17; OR = 1.8, 95% CI 0.8–3.9). **e**, As in **d** but in the ipilimumab-treated subgroup. Tumors with low immune infiltrate scores were strongly associated with PD (infiltrate high: *n* = 10 responders and 7 progressors; infiltrate low: *n* = 1 responder and 9 progressors; two-sided Fisher’s exact test, *P* = 0.02; OR = 12.9, 95% CI 1.3–125.8). **f**, As in **d** but in the ipilimumab-naive subgroup. There was no statistically significant difference between tumors with high and low immune infiltrate scores (infiltrate high: *n* = 14 responders and 15 progressors; infiltrate low: *n* = 17 responders and 18 progressors; two-sided Fisher’s exact test, *P* = 1.0; OR = 0.99, 95% CI 0.4–2.6). **g**, Schematic of the hypothesized effect of ipilimumab treatment on immune response as a predictor of subsequent response to anti-PD1 ICB. Tumors that were treated with ipilimumab but failed to have an immune response or infiltrate in the tumor microenvironment were strongly predicted to have intrinsic resistance to anti-PD1 ICB. However, having an immune response did not guarantee a subsequent response to anti-PD1 ICB. In ipilimumab-naive tumors, neither the presence nor the absence of immune infiltrate was a good predictor of anti-PD1 ICB response.

To further dissect the impact of MHC-II expression on patient response, we stratified the cohort into patients with high and low MHC-II expression (ssGSEA, median split). In the overall cohort, low MHC-II expression was associated with primary resistance (Fig. 4a; Fisher’s exact test, *P* = 0.01; OR = 2.9, 95% confidence interval (CI) 1.3–6.5), but this association was largely driven by the ipilimumab-treated subgroup (Fig. 4b; Fisher’s exact test, *P* = 0.02; OR = 9.9, 95% CI 1.5–63.7), with a nonsignificant association in the ipilimumab-naive subgroup (Fig. 4c; Fisher’s exact test, *P* = 0.32; OR = 1.9, 95% CI 0.7–5.1). A formal interaction test of previous ipilimumab treatment status with MHC-II expression for predicting response was consistent with a subgroup-specific effect of low MHC-II expression on the ipilimumab-experienced subgroup (OR = 0.20, 95% CI 0.02–1.64), although this was nonsignificant (*P* = 0.13) in this small cohort.

A similar analysis of estimated total immune infiltrate levels^41^ (with tumors split by the median into high and low groups) showed that low immune infiltrate was significantly associated with intrinsic resistance in the ipilimumab-treated subgroup (Fig. 4e; Fisher’s exact test, *P* = 0.02; OR = 12.9, 95% CI 1.3–125.8), but not in the ipilimumab-naive subgroup (Fig. 4f; Fisher’s exact test, *P* = 1.00; OR = 0.99, 95% CI 0.4–2.6) or overall cohort (Fig. 4d; Fisher’s exact test, *P* = 0.17; OR = 1.8, 95% CI 0.8–3.9), indicating a subgroup-specific association in the ipilimumab-experienced subgroup of low immune infiltrate with resistance to therapy (OR = 0.08, 95% CI 0.007–0.97; *P* = 0.047).

Taken together, these findings suggest that evidence of immune response in the tumor microenvironment at the time of progression following anti-CTLA4 ICB is a necessary but not sufficient marker for response to anti-PD1 ICB therapy; patients without immune response to anti-CTLA4 ICB are very likely to also be intrinsically resistant to anti-PD1 ICB, highlighting a high-risk and poor-prognosis subgroup of patients (Fig. 4g).

### Integrative predictive modeling of primary resistance

**Extended Data Fig. 7:**
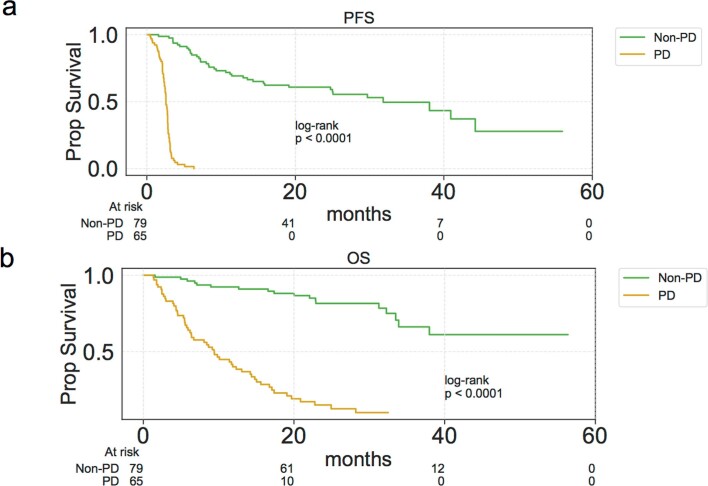
Survival of patients stratified by progressive disease (PD) or non-PD (SD, MR, PR, or CR) best response (RECIST 1.1) to PD-1 ICB. (**a**) PFS (two-sided logrank p = 4.0e-38 (**b**) OS (two-sided logrank p = 1.2e-19).

Patients with primary resistance to anti-PD1 ICB have poor survival (Extended Data Fig. 7), and the ability to predict these patients would enable individualized management regimens (for example combination ICB) to improve outcomes. Thus, we set out to develop parsimonious predictive models integrating clinical, genomic and transcriptomic features to predict PD (primary resistance) versus non-PD (CR, PR, SD and MR) and developed separate predictive models in ipilimumab-treated and ipilimumab-naive subgroups.

**Fig. 5:**
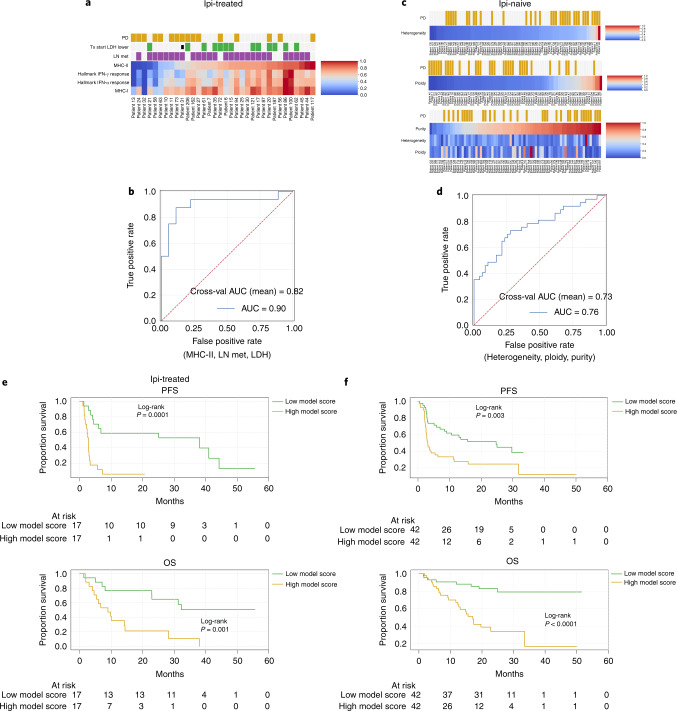
Integrative predictive modeling of intrinsic resistance to anti-PD1 ICB. **a**, CoMut plot showing the relationship between response and predictive features in the ipilimumab-treated subgroup. Each column represents a patient, and the top row indicates whether the patient had PD (intrinsic resistance) or non-PD (CR, PR, SD or MR) as the best response. Patients are sorted by MHC-II HLA score, which was the most predictive feature and was correlated with MHC-I and IFN response pathway scores. MHC-II HLA, LDH at treatment initiation and the presence of lymph node metastases (LN met) were features used for our logistic regression model, chosen using forward selection (Methods). **b**, A receiver–operator characteristic (ROC) curve for our predictive model for ipilimumab-treated tumors (*n* = 34 patients) using MHC-II HLA, LDH and lymph node metastases as features. The AUC was 0.9, and the log-likelihood ratio was *P* = 0.0003. The fivefold cross-validation mean AUC was 0.83. **c**, CoMut plot showing the relationship between response and predictive features in the ipilimumab-naive subgroup. Each column represents a patient, and the top row indicates whether the patient had PD (intrinsic resistance) or non-PD as the best response. Patients are sorted by tumor heterogeneity (top), tumor ploidy (middle) and tumor purity (bottom), which were the three features chosen in our predictive model (Methods). Purity, ploidy and heterogeneity were independent predictors in the multivariate model (Supplementary Table 9). **d**, The ROC curve for our predictive model for ipilimumab-naive tumors (*n* = 84 tumors) using heterogeneity, purity and ploidy as features. The AUC was 0.77, and the log-likelihood ratio was *P* = 0.0003. The average tenfold cross-validation mean AUC was 0.73. **e**, Survival (PFS and OS as indicated) stratified by high versus low predictive model score (split by the median) in ipilimumab-treated tumors in our discovery cohort. Tumors with high scores had worse PFS and OS (two-sided KM log-rank test, *P* = 0.0001 and *P* = 0.001, respectively). **f**, Survival (PFS and OS as indicated) stratified by high versus low predictive model score (split by the median) in ipilimumab-naive tumors in our discovery cohort. Tumors with high scores had worse PFS and OS (two-sided KM log-rank test, *P* = 0.003 and *P* = 6.3 × 10^−5^, respectively).

**Extended Data Fig. 8:**
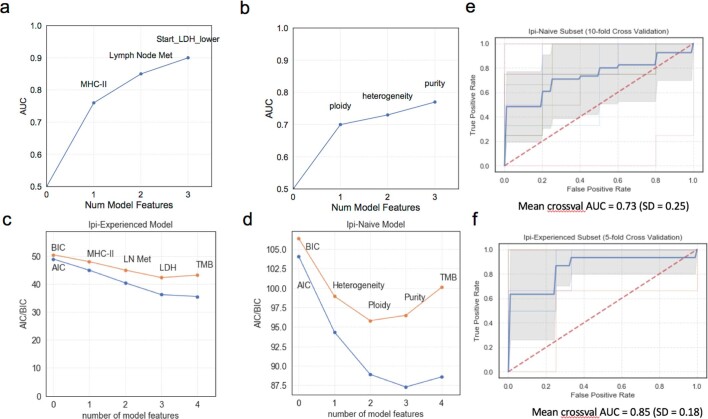
Model selection criteria with increasing number of features by forward selection; cross-validation AUC distribution of final models. (**a**) Model AUC for the ipi-treated subgroup. Each point is labeled with the additional feature, and the final model includes MHC-II, Lymph node metastasis, and LDH below the median as features. (**b**) Model AUC for the ipi-naive subgroup. Each point is labeled with the additional feature, and the final model includes ploidy, heterogeneity, and purity as features. (**c**) Model AIC/BIC for the ipi-experienced subgroup. (**d**) Model AIC/BIC for the ipi-naïve subgroup. (**e**) Using 10-fold crossvalidation in the ipilimumab-naïve subset, mean crossvalidation AUC is 0.73, with a SD of 0.25. Each fold split the cohort into a training (n = 74 patients) and test set (n = 10 patients) ((**f**) In the ipi-experienced subset, mean crossvalidation AUC is 0.85, with a SD of 0.18. Each fold split the cohort into a training (n = 27 patients) and test set (n = 7 patients).

In the ipilimumab-treated group (*n* = 34 with WES and RNA-seq), there were 16 patients who had PD and 18 who had non-PD. Using a forward-selection approach to choose the features of a parsimonious predictive model (Methods), low MHC-II HLA expression was most strongly predictive of PD (Fig. 5a) and was correlated with MHC-I HLA, IFN-α and IFN-γ response pathway scores. In the final multivariate model, high MHC-II expression, low lactate dehydrogenase (LDH; below the median of 247 U l^−1^) and the presence of lymph node metastases were independent predictors of non-PD (*P* = 0.03, *P* = 0.02 and *P* = 0.04, respectively, Supplementary Table 8 and Extended Data Fig. 8a,c), and the model had an area under the curve (AUC) of 0.90 in our discovery cohort (fivefold cross-validation mean AUC = 0.83; empiric *P* < 0.001; Fig. 5b, Extended Data Fig. 8f and Methods). Notably, TMB did not significantly improve model fit (log-likelihood ratio, *P* = 0.10), was not an independently predictive feature (*P* = 0.18) and did not meet Bayesian information criteria (BIC; Extended Data Fig. 8c) when added to the model.

**Extended Data Fig. 9:**
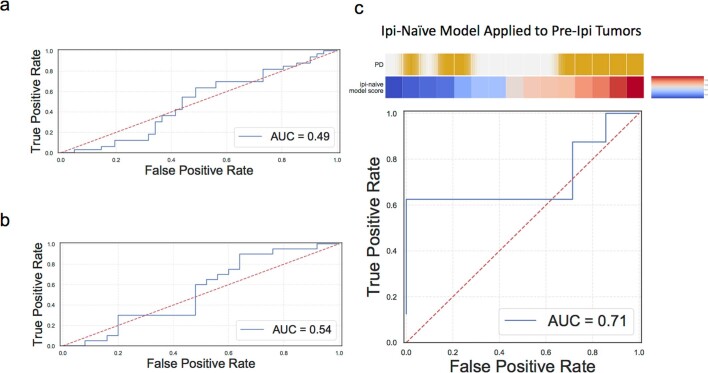
Subgroup Model ROC curves applied to other subgroups. (**a**) Ipilimumab-treated subgroup model applied to ipilimumab-naive (n = 74 patients) subgroup; (**b**) Ipilimumab-naive model applied to ipilimumab-treated (n = 45 patients) subgroup; (**c**) Ipi-naïve model predicting PD as best response to PD-1 ICB applied to pre-ipilimumab-treated (n = 15 patients) tumors. (top) Comut plot overlaying best response and the ipi-naïve model score. Each column is one of 15 ipilimumab-treated tumors biopsied prior to ipilimumab therapy, ordered by the predictive model score. The 5 highest scoring tumors were all PD. (bottom) Receiver-operator curve for our ipilimumab-naive predictive model applied to pre-ipilimumab tumors (n = 15), with an AUC of 0.71.

In the ipilimumab-naive group, there were 34 patients with PD and 41 with non-PD. In the multivariate predictive model, higher heterogeneity, lower ploidy and higher purity were independently predictive of PD (*P* = 0.025, *P* = 0.014 and *P* = 0.046, respectively; Fig. 5c, Supplementary Table 9 and Extended Data Fig. 8b,d), with an AUC of 0.77 (tenfold cross-validation mean AUC of 0.73; empiric *P* = 0.036; Fig. 5d, Extended Data Fig. 8e and Methods). TMB did not significantly improve the model fit (log-likelihood ratio test, *P* = 0.63), was not an independently predictive feature (*P* = 0.63) and did not meet BIC criteria (Extended Data Fig. 8d) when added to this multivariate model. Further, each model’s performance was specific to its subgroup (*P* = 0.004 and *P* = 0.018 for the interaction between ipilimumab-experienced and ipilimumab-naive model scores, respectively, and previous ipilimumab therapy). Each model had poor performance when applied to the opposing subgroup (AUC = 0.49 and AUC = 0.54, respectively; Extended Data Fig. 9a,b), suggesting that previous ipilimumab treatment status may stratify the appropriate predictors and predictive models to be applied in these subgroups.

**Extended Data Fig. 10:**
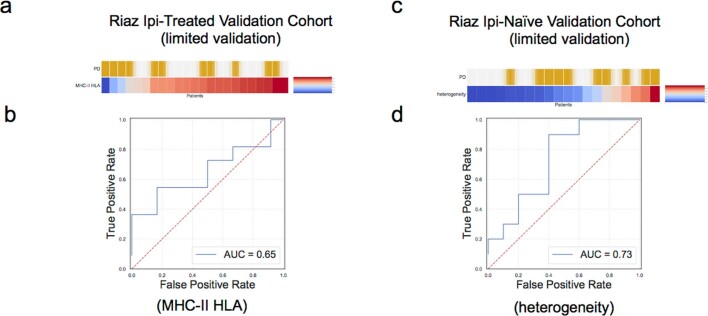
Limited validation testing single feature models in validation cohort. (**a**) Relationship between response and MHC-II HLA score in the ipilimumab-treated subgroup of a validation cohort. Each column is a patient, and patients are sorted by MHC-II HLA score. (**b**) Validation of Predictive Model for ipilimumab-treated tumors. In a validation cohort (n = 23), a model using the only common available feature (MHC-II HLA score) had an AUC of 0.65 (log likelihood ratio p = 0.09, empiric p = 0.21, Methods). (**c**) Relationship between response and MHC-II HLA score in the ipilimumab-treated subgroup of a validation cohort. Each column is a patient, and patients are sorted by MHC-II HLA score. (**d**) Validation of Predictive Model for ipilimumab-naive Tumors. In a validation cohort (n = 20), a model using the only common available feature (heterogeneity) had an AUC of 0.73 (log likelihood ratio p = 0.17, empiric p = 0.066, Methods).

We attempted to validate our models in independent cohorts but were limited by a lack of publicly available cohorts with all the molecular features and relevant clinical data on previous ipilimumab therapy and biopsy timing used in our integrated model. In a limited validation, we tested predictive models incorporating individual features where data were available in an independent validation cohort^44^ (Methods), and found concordant predictions of primary resistance with low MHC-II HLA expression in ipilimumab-treated tumors and higher heterogeneity in ipilimumab-naive tumors (Extended Data Fig. 10), although neither of these predictors was significant in this small cohort (empiric *P* = 0.21 and *P* = 0.066, respectively; Methods).

In an exploratory analysis, we predicted PD in response to anti-PD1 ICB in tumors where the tumor biopsy was taken before ipilimumab treatment (*n* = 15; Fig. 3a) using our ipilimumab-naive predictive model. Of the eight tumors with PD in response to anti-PD1 ICB, five had the highest model scores (Extended Data Fig. 9c), with an overall AUC of 0.71. Interestingly, of the three poorly discriminated tumors with low model scores but PD responses, one (patient 82) was from a brain metastasis and one (patient 80) was an acral melanoma.

Finally, splitting the cohort into subsets with high and low model scores (split by the median), we found large differences in OS and PFS in both the ipilimumab-treated subgroup (Fig. 5e; median PFS: 38.1 months versus 2.8 months, log-rank *P* = 0.0001; median OS: unreached versus 9.0 months, log-rank *P* = 0.001) and ipilimumab-naive subgroup (Fig. 5f; median PFS: 24.7 months versus 3.1 months, log-rank *P* = 0.005; median OS: unreached versus 15.0 months, log-rank *P* < 0.0001).

## Discussion

In this study, we analyzed a uniformly clinically annotated cohort of patients with advanced melanoma treated with anti-PD1 ICB monotherapy for whom WES and RNA-seq data were available. While we observed an association between response and TMB, this observation was confounded by disease subtype, strongly suggesting that TMB cannot be applied generically across melanoma subtypes as a predictive biomarker for anti-PD1 ICB.

Beyond TMB, we found that MHC-II expression, tumor heterogeneity, purity and ploidy were associated with ICB response. In two previous studies^47,48^, MHC-II expression on tumor cells by immunohistochemistry was found to be predictive for response to anti-PD1 ICB and was hypothesized to represent a subset of tumors that could stimulate CD4^+^ helper T cell or cytotoxic activity. Consistent with this hypothesis, we found that MHC-II transcriptomic expression was correlated with expression of *CD4* and the cytolytic molecules *PRF1* and *GZMA* in our cohort. However, whether MHC-II expression represents expression on tumor cells or antigen-presenting cells within the tumor microenvironment cannot be determined from our bulk transcriptome data, and whether the association of PD1 ICB response with MHC-II expression is limited to tumor-cell-specific MHC-II expression is unclear. Notably, CD8^+^ T cell markers were not higher in responders versus progressors in our cohort, and although MHC-II, MHC-I, IFN-γ and IFN-α response pathway expression was correlated, MHC-II expression was the best predictor of response in our cohort. Further, we found evidence for the involvement of other immune compartments (for example, B cell markers enriched in ipilimumab-experienced responders) in ICB response, consistent with data from a recent trial demonstrating higher B cell infiltrate in responders to neoadjuvant immunotherapy^46^, although the specific cell types, functional states and tumor immune interactions are not yet well characterized.

Tumor heterogeneity (the proportion of subclonal mutations) has previously been associated with poor prognosis across multiple tumor types and therapies^49–51^. High heterogeneity suggests a highly mutagenic disease and a high degree of subclonality, with a higher likelihood of preexisting or rapidly evolving resistant clones. Interestingly, in our cohort, four patients had a very high TMB with an alkylating chemotherapy mutational signature (and known previous alkylating chemotherapy); low tumor heterogeneity distinguished the two responders from the two nonresponders (with SD and PD as the best response), who had high tumor heterogeneity with a majority of subclonal mutations. This association has also been observed in dacarbazine-experienced patients with melanoma treated with anti-CTLA4 ICB^49^, suggesting that tumor heterogeneity may be significantly correlated with ICB resistance.

In our cohort, higher ploidy and lower purity were associated with ICB response, but the biological basis of these relationships is unclear. Genome doubling events are common in cancer and may accelerate genome evolution by increasing the tolerance of genome instability^52^, and higher aneuploidy has been associated with a worse response to ICB^18,19^. However, whether genome doubling (and higher ploidy) is also associated with increased immunogenicity is unclear. Purity is negatively correlated with expression of markers of immune response and may be a proxy for the level of immune response in the tumor microenvironment in this setting rather than an artifact of tumor sample processing. However, tumor purity may also reflect differences in tumor biology leading to intrinsic resistance.

Notably, we found that previous exposure to anti-CTLA4 ICB affected the predictors of response to anti-PD1 ICB, although patients with and without exposure had similar response rates to anti-PD1 ICB. Immune-related markers are strongly enriched in responders compared to progressors with previous ipilimumab exposure, but this relationship is less clear in ipilimumab-naive patients. Specifically, post-ipilimumab tumors with poor immune response at progression were resistant to further anti-PD1 ICB, whereas ipilimumab-naive immune-poor and immune-infiltrated tumors were similarly likely to respond to anti-PD1 ICB. Whether anti-CTLA4 ICB induces or, alternatively, reveals an immune-resistant state in a subset of melanomas is an important question that deserves further evaluation. Further, cross-resistance to sequential ICB may also predict resistance to simultaneous combination ICB; this hypothesis should be evaluated in an appropriate cohort.

Building on these findings, we constructed predictive models integrating clinical, genomic and transcriptomic characteristics to identify patients with melanoma with intrinsic resistance to anti-PD1 ICB. Integrating multiple clinical and molecular features resulted in superior discrimination compared to models with any single feature or modality. In patients treated with ipilimumab, low MHC-II expression and high LDH predicted intrinsic resistance, whereas lymph node metastasis predicted improved response to therapy. MHC-II^47,48^ and LDH^53^ have previously been implicated in predicting anti-PD1 responsiveness. Lymph node metastases might provide a reservoir of tumor-specific immune cells, facilitating their activation by physiologic lymph node function; recent experimental data in a murine model suggests that lymph node metastases are necessary for PD1 response^54^, and recent clinical data showing greater tumor-resident T cell clone response to neoadjuvant compared to adjuvant immunotherapy further supports this hypothesis^55^. Similarly, integrating tumor heterogeneity, ploidy and purity for ipilimumab-naive disease resulted in a higher AUC than was obtained with any single-feature model alone. Beyond predicting response, these parsimonious models strongly stratified patients by PFS and OS, suggesting potential clinical applicability in identifying patients at high and low risk.

These findings will require validation in independent and larger cohorts; at the time of our study, limited data were publicly available where molecularly sequenced tumors with previous treatment data and all relevant clinical parameters were available for validation. Further, heterogeneity in sequencing approaches and data normalization between cohorts hindered our ability to develop standardized features to create and validate models. However, our results highlight the value of integrating rich clinical data with molecular tumor characterization and the need to generate such multimodal data.

## Methods

### Patient cohort and clinical end points

Patients were identified in databases of participating sites. For enrollment, patients were required to have advanced melanoma and to have received PD1 blockade as a palliative treatment. Tissue obtained before PD1 blockade was required for enrollment and was collected during routine medical care. Clinicopathological and demographic data were collected from patient records locally and are shown in Table 1. Age, stage and ECOG performance status were documented before the first application of anti-PD1 ICB. LDH was measured within 28 d of the first application of nivolumab or pembrolizumab. OS was defined as the time between the first application of anti-PD1 ICB and the date of death (any cause). For subjects without documentation of death, OS was censored on the last date the patient was known to be alive. BOR to anti-PD1 ICB was assessed according to RECIST criteria v.1.1 by the participating sites. Patients achieving CR or PR as BOR were grouped as responders, whereas patients showing PD as the best response were referred to as progressors. Patients were classified as MR when achieving unequivocal responses in individual existing lesions but also progression in others or new lesions. PFS was defined as the time between the first application of anti-PD1 ICB and the date of documented disease progression. For patients without documentation of progression, PFS was censored on the last date the patient was known to be without progression.

This retrospective study and associated informed consent procedures were approved by the central Ethics Committee (EC) of the University Hospital Essen (12-5152-BO and 11-4715). Approval by the local EC was obtained by investigators if required by local regulations.

### Samples

Samples were collected retrospectively and obtained by excision or biopsy of melanoma tissue, collected locally at the participating sites and provided formalin-fixed and paraffin-embedded (FFPE). Samples were collected between January 2013 and June 2016. The median time from biopsy to initiation of anti-PD1 blockade was 2.1 months with 90% of samples being collected 6 months before the first application of nivolumab or pembrolizumab. All biopsies were from metastatic sites, with the exception of eight biopsies; seven were from a primary lesion and one was from a recurrence at a primary site, representing less than 6% of the overall cohort.

### Whole-exome and whole-transcriptome sequencing

DNA extraction, whole exome library preparation and sequencing were performed for samples as previously described^10,23^. Slides were cut from FFPE blocks and macrodissected for tumor-enriched tissue. Paraffin was removed from FFPE sections and cores using CitriSolv (Fisher Scientific), followed by ethanol washes and tissue lysis overnight at 56 °C. Samples were then incubated at 90 °C to remove DNA cross links. Extraction of DNA, and, when possible, RNA was performed using the QIAGEN AllPrep DNA/RNA mini kit (51306). Germline DNA was obtained from peripheral blood mononuclear cells and adjacent normal tissue.

Whole-exome capture libraries were constructed from 100 ng of DNA from tumor and normal tissue after sample shearing, end repair and phosphorylation and ligation to barcoded sequencing adaptors. Ligated DNA was size selected for lengths of 200–350 bp and subjected to exonic hybrid capture using Illumina library preps. The sample was multiplexed and sequenced using Illumina HiSeq technology. The Illumina exome sequencing approach uses Illumina’s in-solution DNA-probe-based hybrid selection method that applies principles similar to those of Broad Institute–Agilent Technologies’ in-solution RNA-probe-based hybrid selection method^57,58^ to generate Illumina exome sequencing libraries.

Total RNA was assessed for quality using the Caliper LabChip GX2. The percentage of fragments with a size greater than 200 nucleotides (DV200) was calculated using software. An aliquot of 200 ng of RNA was used as the input for first-strand cDNA synthesis using Illumina’s TruSeq RNA Access Library Prep kit. Synthesis of the second strand of cDNA was followed by indexed adaptor ligation. Subsequent PCR amplification enriched for adaptor-ligated fragments. The amplified libraries were quantified using an automated PicoGreen assay.

A total of 200 ng of each cDNA library, not including controls, was combined into four-plex pools. Capture probes that target the exome were added and hybridized for the recommended time. Following hybridization, streptavidin magnetic beads were used to capture the library-bound probes from the previous step. Two wash steps effectively removed any nonspecifically bound products. These same hybridization, capture and wash steps were repeated to assure high specificity. A second round of amplification enriched the captured libraries. After enrichment the libraries were quantified with qPCR using the KAPA Library Quantification kit for Illumina sequencing platforms and were then pooled at an equimolar ratio. The entire process was in a 96-well format and all pipetting was performed using Agilent Bravo or Hamilton Starlet.

Pooled libraries were normalized to 2 nM and denatured using 0.2 N NaOH before sequencing. Flowcell cluster amplification and sequencing were performed according to the manufacturer’s protocols using either the HiSeq 2000 v.3 or HiSeq 2500. Each run generated 76-bp paired-end reads with a dual eight-base index barcode. Data were analyzed using the Broad Picard Pipeline, which includes demultiplexing and data aggregation.

### Quality control and variant calling

Initial exome sequence data processing and analysis were performed using pipelines at the Broad Institute. After alignment from the Broad Picard Pipeline, BAM files were uploaded into the Firehose infrastructure (https://software.broadinstitute.org/cancer/cga/Firehose), which managed intermediate analysis files executed by analysis pipelines. Sequencing data were incorporated into quality-control modules in Firehose to compare the tumor and normal genotypes and ensure concordance between samples. Quality-control cutoffs were as follows: mean target coverage >50× (tumor) and >20× (matched normal), cross-contamination of samples estimation (ContEst^59^) <5% and tumor purity ≥10% (Extended Data Fig. 1).

#### Power calculation quality control

To limit our analysis to samples where we had adequate power to call somatic variants, we performed a downstream per-sample power calculation. For each sample, we performed a Monte Carlo simulation of 1,000 true clonal mutations using the following procedures:

- Sample the number of reads from the sample-specific coverage distribution
- Draw the number of tumor reads from a binomial distribution using the estimated tumor purity
- Draw the number of mutation reads from a binomial distribution given the assumption of a heterozygous mutation and no copy number variation
- Characterize the mutation as detected or not on the basis of a log odds threshold of 6.3 (consistent with the MuTect^60^ implementation).

The estimated power to detect clonal mutations is the proportion of simulated mutations detected (for example, 800 detected out of 1,000 simulated clonal mutations is 80% power), which is a function of both sample-specific sequencing depth of coverage and tumor purity. Three tumors were excluded using this threshold (Extended Data Fig. 1).

#### Variant calling

The MuTect algorithm^60^ was applied to identify somatic single-nucleotide variants in targeted exons, with computational filtering of artifacts introduced by DNA oxidation during sequencing^61^ or FFPE-based DNA extraction using a filter-based method. Strelka^62^ was applied to identify small insertions or deletions. Identified alterations were annotated using Oncotator^63^.

#### TMB and neoantigen load

For purposes of analysis, the TMB was calculated as the log of the number of nonsynonymous mutations detected from WES. Mutations per Mb was calculated by dividing the total number of nonsynonymous mutations by the number of bases with sufficient coverage in the tumor and normal samples (≥14× and ≥8×, respectively) to call mutations (https://software.broadinstitute.org/cancer/cga/mutect). Neoantigen prediction was performed as previously described^10^. Briefly, HLA type was inferred using POLYSOLVER^64^, which uses a Bayesian classifier to determine the genotype of each patient. Neoantigens were predicted for each patient by defining all novel nine and ten amino-acid sequences resulting from mutations and determining whether the predicted binding affinity to the patient’s germline HLA alleles was <500 nM using NetMHCpan (v.2.4)^65^.

#### Copy number variants

The total number of copy number alterations for individual tumors was inferred using adaptations of a binary segmentation algorithm^66^ (CapSeg) comparing fractional exon coverage for tumor segments to a panel of normal samples, generating exomic segments and segment copy number. Copy number data were inspected visually and manually for focal amplifications and deletions and genes were annotated with Oncotator^63^. For allelic copy number alterations, heterozygous single-nucleotide polymorphisms were identified and integrated into the binary segmentation algorithm (Allelic CapSeg) and allelic segments were further adjusted for tumor purity and ploidy using estimates derived from ABSOLUTE^67^. We then called allelic amplifications and deletions, following previously described methodology and criteria^68^ integrating segment focality and the purity- and ploidy-corrected allelic copy number.

#### Purity and ploidy

Purity and ploidy were estimated using the ABSOLUTE algorithm^67^, which integrates variant allele frequency distributions and copy number variants to estimate absolute tumor purity and ploidy and infer the cancer cell fraction, the proportion of cancer cells in the sample that contain each mutation. Allelic segments following purity and ploidy correction were used to estimate allelic copy number.

#### Heterogeneity and aneuploidy

Heterogeneity was estimated as the proportion of mutations in each sample that were inferred to be subclonal. Clonal mutations were defined as having a cancer cell fraction ≥0.8, while other mutations were defined as subclonal; we chose this definition as a simple conservative approach with high specificity.

To estimate aneuploidy, we used the proportion of the genome inferred to have an allelic amplification or deletion, using the allelic segmentation described above.

#### Mutational signatures

De novo mutational signatures were generated in this cohort using an adaptation of non-negative matrix factorization^69^ via the Brunet update method^70^, as previously described in detail^51^, with the R package SomaticSignatures^71^ and non-negative matrix factorization^72^. Cosine similarity was used to compare the discovered signatures to the 30 existing discovered and validated signatures in COSMIC^25,73^, with a threshold of 0.85, and we also manually visualized and inspected similarities in mutational motifs between our signatures and COSMIC signatures.

#### Transcriptomic analysis

Whole-transcriptome sequencing data from FFPE tissues were aligned using STAR^74^ and quantified with RSEM^75^ to yield gene-level expression in transcripts per million (TPM). For RNA-seq quality control, sequencing- and alignment-specific metrics were considered for each sample. The following alignment metrics (output by the STAR alignment method) were considered: percentage of uniquely mapped reads, average mapped read length, number of splices, mismatch rate per base, percentage of multimapped reads, percentage of reads mapped to too many locations, percentage of unmapped reads due to too many mismatches, percentage of unmapped reads due to reads being too short and percentage of unmapped reads due to other reasons. Additionally, we considered the raw number of reads, the average read length, the read duplication rate and DV200 for each sample. Samples were clustered across quality-control metrics using principal-component analysis, and outlier samples were manually evaluated and discarded. Three samples were removed owing to poor quality: patient 143 was excluded owing to an abnormally low absolute number of reads (number of reads <1 million); patient 107 was excluded owing to an abnormally high percentage of reads mapped to too many locations (>10% of reads), likely indicating high numbers of short or degraded reads; and patient 61 was excluded owing to multiple aberrant quality-control metrics resulting in overall poor quality when considering all metrics in aggregate as well as an aberrant expression profile compared to all other samples. Only transcriptomes from tumors whose WES also passed quality control were included; the final patient cohort for RNA-seq analysis included *n* = 121 transcriptomes.

We excluded certain classes of noncoding genes that constituted a large (>10%) proportion of TPM in the majority of samples. Specifically we excluded genes characterized as snoRNA, using biomaRt^76^ to download Ensembl biotype annotations (using the dataset ‘hsapiens_gene_ensembl’ and version ‘GRCh38.p10’) and excluding genes whose biotype was ‘snoRNA’ (*n* = 380 genes). We then regenerated a new TPM metric for each sample to normalize the total transcriptome sum to 1 million.

For analysis, only genes with TPM > 0 in 25% or more of the samples were included. This excluded 6,158 genes, with 20,848 genes passing this threshold.

#### GSEA and ssGSEA

GSEA^32^ was performed using the Cancer Hallmarks gene sets^33^ from MSigDB at https://cloud.genepattern.org/. We used default settings with 10,000 gene set permutations to generate *P* and *q* values, and we compared progressors and responders in the overall cohort, the ipilimumab-treated subgroup and the ipilimumab-naive subgroup separately.

To generate nonparametric gene set scores in individual samples, we generated ssGSEA projections^43^ for gene sets using rank normalization, including the MHC-II HLA genes (*HLA-DMA*, *HLA-DMB*, *HLA-DOA*, *HLA-DOB*, *HLA-DPA1*, *HLA-DPB1*, *HLA-DQA1*, *HLA-DQA2*, *HLA-DQB1*, *HLA-DQB2*, *HLA-DRA*, *HLA-DRB1* and *HLA-DRB5*) and MHC-I HLA genes (*HLA-A*, *HLA-B*, *HLA-C*, *HLA-E*, *HLA-F*, *TAP1*, *TAP2* and *B2M*). ssGSEA scores were also generated for Cancer Hallmarks gene sets.

#### Absolute immune infiltrate and immune subsets

Estimation of the total immune infiltrate in each sample and immune cell subsets was performed using CIBERSORT with the LM22 gene set^41^ on the CIBERSORT website (http://cibersort.stanford.edu). The absolute mode was enabled and quantile normalization was disabled using the RNA-seq TPM matrix for the cohort.

A separate immune infiltrate and immune cell subsets score analysis was performed using single-cell-derived signatures, and the methodology used for generating normalized signature scores was as described by Jerby-Arnon and colleagues^42^.

As there is no gold standard for inferring immune infiltrate from bulk RNA-seq data, we chose to use CIBERSORT-inferred values throughout the analysis to allow comparisons with other studies, as CIBERSORT has been widely used (for example, in a pan-TCGA immune landscape analysis^77^).

#### Gene expression signatures

Published gene expression signatures related to immune-checkpoint response were collected from the literature and validated in our cohort^18,19,21,34–40^. Sample-wise scores for these gene signatures were calculated from RNA-seq data using TPM values and following the methodology described in corresponding studies. Genes with unavailable expression data were excluded from calculations of gene signature scores. In two gene signatures^39,40^, genes were incorporated independently (that is, weighted) into the published model, but neither the direction nor the coefficient was available, so these signatures were excluded from evaluation. Differences in immune signature scores between responders (CR and PR) and progressors (PD) across all samples, in the ipilimumab-naive subset and in the ipilimumab-treated subset were tested using the Mann–Whitney *U*-test. The predictive utility of these immune signatures was evaluated with AUC values derived from ROC curves of gene signature scores in the complete cohort, ipilimumab-naive subset and the ipilimumab-treated subset. Results and detailed descriptions of evaluated gene signatures are provided in Supplementary Table 6.

### Analysis

Two primary response comparisons were made: (1) responders (defined as having CR or PR as the best RECIST response) versus progressors (defined as having PD as the best RECIST response) and (2) progressors (defined as having non-PD as the best RECIST response) versus non-progressors.

Statistical tests were performed utilizing the Python *scipy.stats* package. To compare numeric features between response categories, including transcriptome expression, a nonparametric MWW rank-sum test (*mannwhitneyu*() function) was used to minimize the effects of outliers. For comparison of the proportion between response categories, a chi-squared test (*chi2_contingency*() function) was utilized. For association of binary variables (for example, association of gene alteration with responders versus progressors), a Fisher’s exact test (*fisher_exact*() function) was utilized to generate a *P* value. A conservative adjusted OR was generated by repeating the Fisher’s exact test, adding one to both the number of gene-mutant responders and progressors. All tests were two sided unless otherwise indicated.

Survival analyses were performed utilizing the Python *lifelines* package^78^. For Kaplan–Meier curve survival analysis, a log-rank test (*logrank_test()* function) was used to compare survival curves.

Hierarchical clustering was performed using the *clustermap()* function from the Python *seaborn* package^79^, with default settings including a Euclidean distance metric and the ‘single’ method of calculating cluster distance (minimization of the nearest point between clusters).

### Validation

For validation, we reviewed the literature and found three studies^18,20,44^ of advanced melanoma treated with anti-PD1 ICB with response, WES and RNA-seq data. However, one did not have information on previous ipilimumab treatment^20^, and another^18^ had only two patients who were naive to ipilimumab and nine who were treated with ipilimumab with post-ipilimumab tumor biopsies and available WES and NanoString data; thus, we used the remaining cohort^44^ as our primary validation cohort.

To allow appropriate validation, only cutaneous, occult, acral and mucosal samples were included from validation cohorts; specifically, uveal and ocular melanomas were excluded (Riaz cohort, *n* = 5 excluded). Only patients with evaluable response criteria were included (Riaz cohort, *n* = 2 excluded). WES, transcriptomic and heterogeneity data were obtained from https://github.com/riazn/bms038_analysis. Fragments per kilobase of transcript per million mapped reads values were converted to TPM to be consistent with our cohort normalization.

### Predictive model generation and cross-validation

We used logistic regression for our model to predict PD as the best RECIST response versus non-PD rather than responder versus progressor to better reflect the real-world setting where all outcomes (PD, SD, MR, PR and CR) are possible. We evaluated genomic, transcriptomic and clinical features. Categorical features were converted to binary features for each categorical value. To be conservative, no gene-level mutations or expression values were individually considered. Global genomic tumor characteristics such as TMB, purity, ploidy, heterogeneity and aneuploidy were considered. Features were generated from the transcriptome, including ssGSEA values for gene sets representing Cancer Hallmarks pathways, and MHC-II and MHC-I antigen-presentation genes, as well as gene expression signatures following the methodology in the respective publications, as described above and in Supplementary Table 6. Clinical characteristics including LDH and ECOG at the start of anti-PD1 ICB, the number of metastatic organs, sex, M stage, the number of different metastatic sites, metastatic sites and melanoma subtype were evaluated (Supplementary Table 1). Features were chosen in a forward-selection-based process, where features that were significantly predictive (*P* < 0.05) when added to the base model were ranked on the basis of the ability of the combined model to discriminate outcomes (using ROC curve AUC as the metric), and the best feature was chosen to be added to the base model. Potential features were evaluated on the basis of a manual review considering biological interpretability and clinical applicability. This process was iterated with the new base model and stopped when no features under consideration were significantly predictive.

The set of tumors with both WES and RNA-seq data was smaller than the set of tumors with only WES data; when the features chosen in model development for ipilimumab-naive tumors resulted in only WES features being chosen, model development was repeated in the superset of tumors requiring only clinical and WES data, and this model in the larger set is reported in the main text.

To estimate the ‘out-of-bag’ AUC, we used *k*-fold cross-validation (splitting the dataset into *k-*subsets, training on *k* − 1 subsets and calculating AUC on the holdout subset) and calculated the mean cross-validation AUC. Given the partially manual review of features, feature selection was not included in cross-validation. For the ipilimumab-treated subset (*n* = 34), we chose *k* = 5 folds, and for the larger ipilimumab-naive subset (*n* = 85), we chose *k* = 10 folds, to maintain a cross-validation holdout set of >5 tumors. Cross-validation scores were calculated using the *cross_val_score* function from the Python *sklearn* package.

To further evaluate the statistical support for our models, we calculated the Akaike information criteria and BIC of each subsequent model after adding an additional feature in forward selection in the ipilimumab-experienced and ipilimumab-naive subgroups (Extended Data Fig. 8c,d), and we also evaluated the addition of TMB as an additional feature to the selected models.

### Permutation testing

To test the statistical significance of differences in FDR *q* values of enriched pathways between ipilimumab-naive versus ipilimumab-experienced subgroups, we performed a permutation analysis. Briefly, we shuffled the ipilimumab-experienced and ipilimumab-naive labels for each tumor, keeping each subgroup size the same and keeping the same number of responders and progressors in each subgroup, and we reran GSEA on each new simulated subgroup. We repeated this 1,000 times to generate a distribution of enriched pathways in each subgroup under the null hypothesis of no relationship between subgroup and pathway enrichment. We then compared our observed outcome within this null distribution to generate an empiric *P* value. For example, for each pathway enriched in ipilimumab-experienced patients, the proportion of simulations with a difference in log *q* value between ipilimumab-experienced and ipilimumab-naive subgroups (equivalent to the ratio of *q* values) greater than or equal to our observed difference, and with an FDR *q* value in the ipilimumab-experienced subgroup equal to or more extreme than our observed ipilimumab-experienced *q* value would represent the empiric *P* value.

We performed a similar permutation test to generate an empiric *P* value for the predictive models for ipilimumab-experienced and ipilimumab-naive subgroups. Briefly, we permuted the outcome labels (progressors and non-progressors) within each subgroup, and generated an AUC and cross-validation AUC for the predictive model with the specified features (that is, MHC-II, LDH and lymph node metastasis for ipilimumab-experienced tumors; purity, ploidy and heterogeneity for ipilimumab-naive tumors) to generate a null distribution of AUCs and cross-validation AUCs under the null hypothesis that these predictors are not associated with outcomes. By permuting the phenotype rather than the predictors, we preserved the inter-predictor structure. Then, the proportion of simulations with AUC and cross-validation AUC greater than our observed AUC represented an empiric *P* value.

### Reporting Summary

Further information on research design is available in the Nature Research Reporting Summary linked to this article.

## Online content

Any methods, additional references, Nature Research reporting summaries, source data, extended data, supplementary information, acknowledgements, peer review information; details of author contributions and competing interests; and statements of data and code availability are available at 10.1038/s41591-019-0654-5.

## Supplementary information

Reporting Summary

Supplementary Data 1 Gene-level mutation calls; copy number alterations; immune cell signatures; mutational signatures; and ssGSEA signatures using Hallmarks gene sets.

Supplementary Data 2 RNA-seq TPM matrix.

Supplementary Tables Supplementary Tables 1–9.

## Data Availability

All reasonable requests for raw and analyzed data and materials will be promptly reviewed by the senior authors to determine whether the request is subject to any intellectual property or confidentiality obligations. Patient-related data not included in the paper may be subject to patient confidentiality. Any data and materials that can be shared will be released via a material transfer agreement. All analyzed sequencing data are in supplementary tables or data available online. Raw sequencing data are available in dbGaP (accession number phs000452.v3.p1).

## Code availability

Python code is available in packages as described in the manuscript. Code to regenerate figures using supplementary data and tables is available at GitHub at https://github.com/vanallenlab/schadendorf-pd1. Additional reasonable requests for code will be promptly reviewed by the senior authors to verify whether the request is subject to any intellectual property or confidentiality obligations, and shared to the extent permissible by these obligations.
