# Author Correction: Integrative molecular and clinical modeling of clinical outcomes to PD1 blockade in patients with metastatic melanoma

**DOI:** 10.1038/s41591-020-0975-4

**Published:** 2020-06-19

**Authors:** David Liu, Bastian Schilling, Derek Liu, Antje Sucker, Elisabeth Livingstone, Livnat Jerby-Arnon, Lisa Zimmer, Ralf Gutzmer, Imke Satzger, Carmen Loquai, Stephan Grabbe, Natalie Vokes, Claire A. Margolis, Jake Conway, Meng Xiao He, Haitham Elmarakeby, Felix Dietlein, Diana Miao, Adam Tracy, Helen Gogas, Simone M. Goldinger, Jochen Utikal, Christian U. Blank, Ricarda Rauschenberg, Dagmar von Bubnoff, Angela Krackhardt, Benjamin Weide, Sebastian Haferkamp, Felix Kiecker, Ben Izar, Levi Garraway, Aviv Regev, Keith Flaherty, Annette Paschen, Eliezer M. Van Allen, Dirk Schadendorf

**Affiliations:** 1 Dana-Farber Cancer Institute, Boston, MA USA; 2 Broad Institute of Harvard and MIT, Cambridge, MA USA; 3 Department of Dermatology, University Hospital Würzburg, Würzburg, Germany; 4 Department of Dermatology, University Hospital, Essen, Germany; 5 German Cancer Consortium of Translational Cancer Research, German Cancer Research Center, Heidelberg, Germany; 6 Harvard Medical School, Boston, MA USA; 7 Skin Cancer Center Hannover, Department of Dermatology and Allergy, Hannover Medical School, Hannover, Germany; 8 Department of Dermatology, University Medical Center, Mainz, Germany; 9 Biophysics Program, Harvard University, Cambridge, MA USA; 10 First Department of Medicine, National and Kapodistrian University of Athens, Athens, Greece; 11 Department of Dermatology, University Hospital Zürich, Zürich, Switzerland; 12 Department of Dermatology, University Medical Center Mannheim, Ruprecht-Karl University of Heidelberg, Mannheim, Germany; 13 Skin Cancer Unit, German Cancer Research Center, Heidelberg, Germany; 14 Department of Medical Oncology, The Netherlands Cancer Institute, Amsterdam, the Netherlands; 15 Skin Cancer Center at the University Cancer Centre, Department of Dermatology, Faculty of Medicine, University Hospital Carl Gustav Carus, Technische Universität Dresden, Dresden, Germany; 16 National Center for Tumor Diseases, Dresden, Germany; 17 German Cancer Research Centre, Heidelberg, Germany; 18 Department of Dermatology, Medical Center-University of Freiburg, Faculty of Medicine, University of Freiburg, Freiburg, Germany; 19 Medizinische Klinik III, Klinikum Rechts der Isar, Technische Universität München, Munich, Germany; 20 Department of Dermatology, University Medical Center Tübingen, Tübingen, Germany; 21 Department of Dermatology, University Hospital Regensburg, Regensburg, Germany; 22 Department of Dermatology, University Hospital Berlin, Berlin, Germany; 23 Eli Lilly and Co., Indianapolis, IN USA; 24 Massachusetts General Hospital, Boston, MA USA

**Keywords:** Cancer genomics, Melanoma, Computational biology and bioinformatics

Correction to: *Nature Medicine* 10.1038/s41591-019-0654-5, published online 2 December 2019.

In the version of this article initially published, the sixth author’s surname (Jerby-Amon) was incorrect. The correct surname is ‘Jerby-Arnon’. The error has been corrected in the HTML and PDF versions of the article.

