# Supplementary material for: Integrative molecular and clinical modeling of clinical outcomes to PD1 blockade in patients with metastatic melanoma
Source: Nat Med. 2019 Dec 2;25(12):1916–27. doi: 10.1038/s41591-019-0654-5 (PMC6898788; doi:10.1038/s41591-019-0654-5)
Supplement: Supplementary file 1 — Reporting Summary [file 41591_2019_654_MOESM1_ESM.pdf]

nature research

Corresponding author(s): Dirk Schadendorf, Eliezer M. Van Allen

Last updated by author(s): Oct 1, 2019

# Reporting Summary

Nature Research wishes to improve the reproducibility of the work that we publish. This form provides structure for consistency and transparency in reporting. For further information on Nature Research policies, see Authors & Referees and the Editorial Policy Checklist.

## Statistics

For all statistical analyses, confirm that the following items are present in the figure legend, table legend, main text, or Methods section.

| n/a | Confirmed | |
|---|---|---|
| ☐ | ☒ | The exact sample size (*n*) for each experimental group/condition, given as a discrete number and unit of measurement |
| ☐ | ☒ | A statement on whether measurements were taken from distinct samples or whether the same sample was measured repeatedly |
| ☐ | ☒ | The statistical test(s) used AND whether they are one- or two-sided<br>*Only common tests should be described solely by name; describe more complex techniques in the Methods section.* |
| ☐ | ☒ | A description of all covariates tested |
| ☐ | ☒ | A description of any assumptions or corrections, such as tests of normality and adjustment for multiple comparisons |
| ☐ | ☒ | A full description of the statistical parameters including central tendency (e.g. means) or other basic estimates (e.g. regression coefficient) AND variation (e.g. standard deviation) or associated estimates of uncertainty (e.g. confidence intervals) |
| ☐ | ☒ | For null hypothesis testing, the test statistic (e.g. *F*, *t*, *r*) with confidence intervals, effect sizes, degrees of freedom and *P* value noted<br>*Give P values as exact values whenever suitable.* |
| ☒ | ☐ | For Bayesian analysis, information on the choice of priors and Markov chain Monte Carlo settings |
| ☒ | ☐ | For hierarchical and complex designs, identification of the appropriate level for tests and full reporting of outcomes |
| ☐ | ☒ | Estimates of effect sizes (e.g. Cohen's *d*, Pearson's *r*), indicating how they were calculated |

*Our web collection on statistics for biologists contains articles on many of the points above.*

## Software and code

Policy information about availability of computer code

| | |
|---|---|
| Data collection | All data needed to regenerate figures and results are provided in supplemental information. Raw sequencing data (BAMs) are in the process of being deposited into dbGAP and will be available for re-analysis |
| Data analysis | All analytical techniques are described and cited in the Methods section. Results generated using publically available websites/tools with parameters are described in Methods. Code to regenerate figures will be available in Github.<br>Software used: Mutect (v1.1.6); Strelka (v.1.0.11; ReCapSeg (https://gatkforums.broadinstitute.org/gatk/categories/recapseg)<br>ABSOLUTE (https://software.broadinstitute.org/cancer/cga/absolute);ONCOTATOR (https://software.broadinstitute.org/cancer/cga/oncotator); POLYSOLVER (https://software.broadinstitute.org/cancer/cga/polysolver);NetMHCpan(v2.4);STAR(v2.4.1a);RSEM(v1.2.15)<br>R-packages: SomaticSignatures(v2.6.1); NMF(v0.20.6)<br>Python packages: lifelines (v0.14.6); seaborn(v0.9.0); scipy(v1.1.0); statsmodels(0.9.0)<br>Websites:<br>CIBERSORT (http://cibersort.stanford.edu)<br>TIDE (http://tide.dfci.harvard.edu)<br>GSEA(https://cloud.genepattern.org) |

For manuscripts utilizing custom algorithms or software that are central to the research but not yet described in published literature, software must be made available to editors/reviewers. We strongly encourage code deposition in a community repository (e.g. GitHub). See the Nature Research guidelines for submitting code & software for further information.

## Data

Policy information about availability of data

All manuscripts must include a data availability statement. This statement should provide the following information, where applicable:

- Accession codes, unique identifiers, or web links for publicly available datasets
- A list of figures that have associated raw data
- A description of any restrictions on data availability

A data availability statement is provided, and data for figures are provided in supplemental information

# Field-specific reporting

Please select the one below that is the best fit for your research. If you are not sure, read the appropriate sections before making your selection.

☒ Life sciences ☐ Behavioural & social sciences ☐ Ecological, evolutionary & environmental sciences

For a reference copy of the document with all sections, see nature.com/documents/nr-reporting-summary-flat.pdf

# Life sciences study design

All studies must disclose on these points even when the disclosure is negative.

| | |
|---|---|
| Sample size | No formal sample size calculation was performed for this retrospective cohort study rendering descriptive results. All available patients meeting clinical inclusion criteria (e.g. metastatic melanoma treated with anti-PD1 therapy) who provided proper consents with available tumor tissue for sequencing from participating sites. |
| Data exclusions | Data exclusions are summarized in the consort diagram in Extended Data Figure 1. From 206 patients assessed for eligibility, exclusion reasons included wrong clinical context (n=5); no matched normal available (n=2); missing response data (n=3); uveal melanoma (n=3); tumor-normal SNP mismatch (n=9); insufficient normal input (n=16); insufficient tumor input (n=5); insufficient coverage (n=8); low tumor purity (n=2); high contamination (n=2); low power to call mutations (n=3); sequencing failure (n=4), leaving 144 samples for further analysis. |
| Replication | Independent validation cohorts with all clinical, genomic, and transcriptomic data to replicate modeling results were not available. We performed limited replication of transcriptome based findings in an independent validation cohort (Riaz et al). |
| Randomization | Our study is a retrospective cohort study without randomization. |
| Blinding | Our study is a retrospective cohort study without blinding. |

# Reporting for specific materials, systems and methods

We require information from authors about some types of materials, experimental systems and methods used in many studies. Here, indicate whether each material, system or method listed is relevant to your study. If you are not sure if a list item applies to your research, read the appropriate section before selecting a response.

### Materials & experimental systems

| n/a | Involved in the study | |
|---|---|---|
| ☒ | ☐ | Antibodies |
| ☒ | ☐ | Eukaryotic cell lines |
| ☒ | ☐ | Palaeontology |
| ☒ | ☐ | Animals and other organisms |
| ☐ | ☒ | Human research participants |
| ☐ | ☒ | Clinical data |

### Methods

| n/a | Involved in the study | |
|---|---|---|
| ☒ | ☐ | ChIP-seq |
| ☒ | ☐ | Flow cytometry |
| ☒ | ☐ | MRI-based neuroimaging |

## Human research participants

Policy information about studies involving human research participants

| | |
|---|---|
| Population characteristics | Patients enrolled suffer from advanced melanoma and received a PD-1 blocking agent as palliative therapy. Briefly, 42% were female and 58% were male; 41% received nivolumab and 59% received pembrolizumab. 7% had unresectable Stage III disease and 93% had Stage IV disease. 11% had brain metastases. 95% had ECOG status 0 or 1. |
| Recruitment | Participating sites identified patients in local databases fulfilling inclusion criteria (Advanced melanoma, treated with PD-1 blockade, tumor tissue available); patient populations and participation at participating sites may vary which may affect the |

| | |
|---|---|
| | representativeness of this cohort and generalizability of these results, but genomic characteristics and response rates are similar to other large published studies. |
| Ethics oversight | The study was approved by the Ethics committee of the University Hospital Essen, Germany (12-5152-BO and 11-4715). |

Note that full information on the approval of the study protocol must also be provided in the manuscript.

## Clinical data

Policy information about clinical studies

All manuscripts should comply with the ICMJE guidelines for publication of clinical research and a completed CONSORT checklist must be included with all submissions.

| | |
|---|---|
| Clinical trial registration | Due to the retrospective nature, no registration has been done. |
| Study protocol | For this retrospective study, inclusion criteria were defined as stated in the manuscript and in this form. Briefly, patients were required to suffer from advanced melanoma and to have received PD1 blockade as palliative treatment, and have tissue obtained prior to PD1 blockade. |
| Data collection | Samples were collected from participating sites between January 2013 and June 2016. Clinical and demographic data were obtained by participating sites from medical records. A CONSORT diagram is included in the manuscript. |
| Outcomes | Overall survival (OS) was defined as the time between first application of PD-1 blockade and date of death (any cause). For subjects without documentation of death, OS was censored on the last date the subject was known to be alive. Best overall response (BOR) to PD-1 blockade was assessed according to RECIST 1.1 by the participating sites. Patients achieving complete (CR) or partial responses (PR) as BOR were grouped as "responder", patients achieving disease control (CR, PR or stable disease (SD)) were summarized as "nonPD" while patients showing progressive disease (PD) as best response are referred to as "progressors". Patients were classified as mixed responders (MR) when achieving unequivocal responses in individual existing lesions but also progression in others or new lesions. Progression-free survival (PFS) was defined as the time between first application of PD-1 blockade and date of documented disease progression. For subjects without documentation of progression, PFS was censored on the last date the subject was known to be without progression. |
